# Monkeyflower (*Mimulus*) uncovers the evolutionary basis of the eukaryote telomere sequence variation

**DOI:** 10.1371/journal.pgen.1011738

**Published:** 2025-06-16

**Authors:** Surbhi Kumawat, Askhan Shametov, Liia R. Valeeva, Yoonha Ju, Irene Martinez, Dhenugen Logeswaran, Hongfei Chen, Jenn M. Coughlan, Julian J.-L. Chen, Yao-Wu Yuan, James M. Sobel, Dal-Hoe Koo, Eugene V. Shakirov, Jae Young Choi

**Affiliations:** 1 Department of Ecology and Evolutionary Biology, University of Kansas, Lawrence, Kansas, United States of America; 2 Department of Biological Sciences, Marshall University, Huntington, West Virginia, United States of America; 3 Department of Plant Pathology, Kansas State University, Manhattan, Kansas, United States of America; 4 Department of Biological Sciences, Binghamton University (SUNY), Binghamton, New York, United States of America; 5 School of Molecular Sciences, Arizona State University, Tempe, Arizona, United States of America; 6 Department of Ecology and Evolutionary Biology, Yale University, New Haven, Connecticut, United States of America; 7 Department of Ecology and Evolutionary Biology, University of Connecticut, Storrs, Connecticut, United States of America; University of Cambridge, UNITED KINGDOM OF GREAT BRITAIN AND NORTHERN IRELAND

## Abstract

Telomeres are nucleoprotein complexes with crucial role of protecting chromosome ends. Because of its vital functions, components of the telomere, including its sequence, should be under strong evolutionary constraint. Yet across the tree of life there are numerous examples of telomere sequence variation and the evolutionary mechanism driving this diversification is unclear. Here, we studied the telomeres in *Mimulus* by investigating the noncoding telomerase RNA (TR), which is a core component of the telomere maintenance complex and determines the telomere sequence in eukaryotes. We conducted *de novo* transcriptomics and genome analysis of 18 species, and discovered *Mimulus* has evolved at least three different telomere sequences: (AAACCCT)_*n*_, (AAACCCG)_*n*_, and (AAACCG)_*n*_. We discovered several species with TR duplications, implying functional consequences that could influence telomere evolution. For instance, *M. lewisii* harbored two sequence-divergent TR paralogs while its sister species the paralog had pseudogenized. Nanopore-sequencing and fluorescence *in situ* hybridization indicated *M. lewisii* had a sequence heterogeneous telomere, and Telomeric Repeat Amplification Protocol combined with Terminal Restriction Fragment analysis confirmed the telomerase can use both TR paralogs for telomere synthesis. Interestingly in closely related species *M. cardinalis*, TR was also duplicated and both paralogs were expressed but its telomere consisted of a single telomere repeat. Evolutionary analysis indicated the TR paralogs arose from an ancient duplication, which also underlies the evolutionary origin of multiple *Mimulus* species with divergent telomere sequences. We propose sequence variation in eukaryotic telomeres arises from an evolutionary process involving TR duplication, sequence divergence, and loss of TR paralog.

## Author summary

One of the most fascinating phenomena in evolutionary biology is the rapid evolution of molecular complexes with conserved functions across the tree of life. Studying these complexes has led to novel insights into the function and evolution of universally important molecular systems. This study investigated the evolution of the telomere, a nucleoprotein complex that protects chromosome ends in all eukaryotes. Telomeres have a crucial role in genomic stability and protection, hence components of the telomere complex are thought to be under strong evolutionary constraint. In plants, however, their telomeres have evolved an enormous range of sequence variation for reasons that are largely unknown. Here, we present results from Monkeyflower (*Mimulus*) and propose an evolutionary model to explain why telomere sequence varies in plants. We investigated the evolution of *Mimulus* telomeres by studying the long noncoding telomerase RNA (TR), which is a core component of the telomere maintenance complex and determines the telomere DNA sequence. We posit that complex evolutionary changes in the TR involving transposition-mediated gene duplication results in plant species evolving telomeres with mixed repeat sequences.

## Introduction

The ends of all linear eukaryotic chromosomes need special attention. Since DNA replication is a semiconservative process, without any intervention chromosome ends will progressively erode with each round of DNA replication (*i.e.,* the end replication problem) [1,2]. In addition, naked chromosome ends trigger DNA damage responses in the cell and result in detrimental consequences, such as cellular senescence and chromosomal fusions [3]. To counteract these highly deleterious genome instabilities, chromosome ends are capped by a nucleoprotein complex called the telomere. The telomere is composed of TG-rich microsatellite DNA sequences [4] which are maintained by the telomerase, a ribonucleoprotein enzyme complex that ensures the proper replication of chromosome ends [5]. Additional specialized telomere binding proteins also bind to the telomere and protect chromosome ends from being detected as damaged DNA [6,7].

All eukaryotes with linear chromosomes need the telomere for proper chromosome end protection [8]. Due to this crucial function, telomeres are thought to be under strong evolutionary constraint and resistant to molecular changes. For instance, all vertebrates have an identical telomere repeat motif TTAGGG that is present multiple times at chromosome ends [we symbolize the telomere structure with a nucleotide sequence TTAGGG repeating *n* times at the chromosome end as (TTAGGG)_*n*_] [9]. These data suggests for vertebrates the strong purifying selection has prohibited evolutionary changes in the telomere sequence. However, across the tree of life, vertebrates are rather the evolutionary exceptions as there are numerous animal lineages that have evolved novel telomere sequence types. For instance nematodes have evolved (TTAGGC)_*n*_ and (TTAGAC)_*n*_ telomeres [10] that differs from the (TTAGGG)_*n*_ vertebrate telomeres. In insects, high turnover of telomere sequences are observed within the Dipetera group [11,12] whereas in other invertebrates for example spiders [13], the telomere sequence structures are unknown. Fungi are also well known for their diversity of telomeric sequences, where the repeat sequence content and length are highly variable across numerous fungal lineages [14].

Across the plant kingdom, telomeres also display a diversity of telomere repeat sequences [4,15]. *Arabidopsis thaliana* was the first plant species to have its telomere sequence decoded as (TTTAGGG)_*n*_ [16] and its similarity to animal telomere sequence (only a single nucleotide change) suggests (TTTAGGG)_*n*_ is the likely ancestral telomere sequence in plants. Subsequent studies have then revealed many plant species also harbored the same *Arabidopsis*-type telomere sequence [17]. But there are also multiple plant lineages deviating from the *Arabidopsis*-type telomere repeat, and the consensus sequence can be grouped into three major types: (1) extension or contraction of the thymine or guanine nucleotide [*e.g.,* (TTAGGG)_*n*_ in Asparagales [18], (TTTTTTAGGG)_*n*_ in *Cestrum elegans* [Solanaceae] [19], (AATGGGGGG)_*n*_ in *Cyanidioschyzon merolae* [Cyanidiaceae] [20]], (2) insertion or substitution of non-thymine or -guanine nucleotides [*e.g.,* (TTCAGG)_*n*_ and (TTTCAGG)_*n*_ in *Genlisea* [21]], and (3) large changes with minimal resemblance to the *Arabidopsis*-type sequence [*e.g.,* (CTCGGTTATGGG)_*n*_ in *Allium* [22]]. There are also entire plant clades (e.g., Aquifoliales and Boraginales) that have completely lost the *Arabidopsis*-type telomere repeats [17], indicating that sequence turnover of telomeres occurs commonly within the plant kingdom.

**Fig 1:**
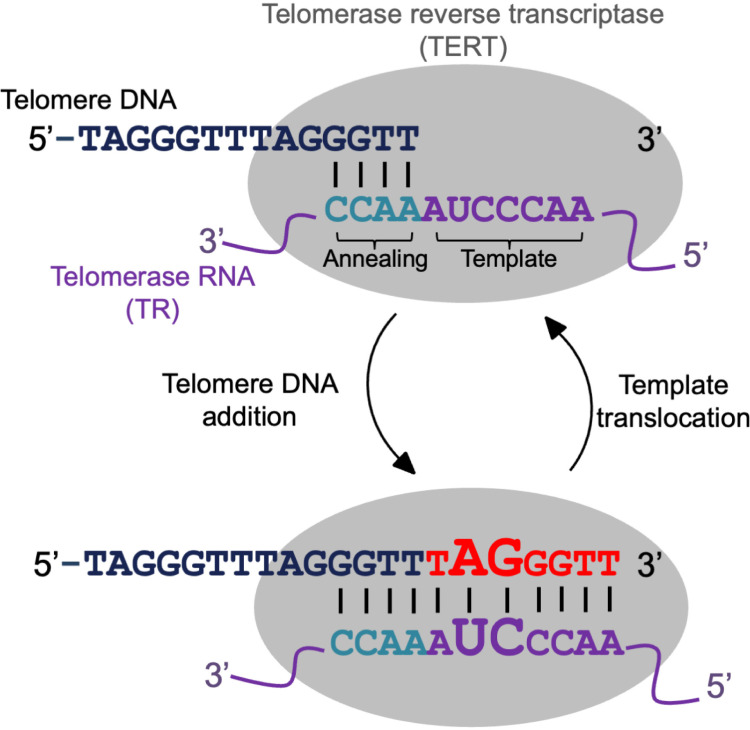
Hypothesized model for the *Mimulus* telomerase activity cycle. Figure was adapted from [32] and the activity model is based on [33]. A major summary of this model is that the TR templating sequence (purple letters) dictates the synthesis of the telomeric DNA sequence (red letter), hence the TR sequence evolution can be studied to understand the biological basis of telomere sequence evolution. The core telomerase ribonucleoprotein complex consists of TERT and TR, and functions to extend chromosomal ends with species-specific telomere DNA sequences. The TR template region harbors two sequences, the annealing sequence (teal letters) and the templating sequence (purple letters). The TR annealing sequence forms a DNA/RNA duplex with the 3’ single-stranded ends of the telomeric DNA. Using reverse transcription, TERT protein then synthesizes the deoxyribonucleotides to the 3’ end of the telomeric DNA (red letters) by using the TR templating sequence as a guide. After reaching the end of the templating sequence the DNA/RNA duplex separates and the TR templating domain slides over and reanneals with the telomeric DNA to open up the templating sequence for the next round of telomere repeat synthesis. The variable telomere DNA sequence that we discovered in this study is highlighted as enlarged letters in the DNA/RNA duplex formed in the TR templating sequence region.

The remarkable diversity of telomere repeat sequence in many animal and plant lineages suggests a possible common evolutionary mechanism might underlie the sequence variation in eukaryotic telomeres. But what evolutionary processes are involved in the eukaryote telomere evolution is largely an open question. This question can be partially answered by studying the telomerase, which functions to maintain telomeres and synthesize telomeric DNA at chromosome ends. Telomerase is comprised of two major components (see Fig 1 for molecular model): 1) the telomerase reverse transcriptase (TERT) that synthesizes the telomere DNA sequences and 2) the telomerase RNA (TR) subunit, a long noncoding RNA gene that serves as a template during DNA synthesis [23,24]. Evolutionarily, TERT is a largely conserved protein across the eukaryotic kingdom [25]. The TR, on the other hand, is a rapidly evolving sequence, displaying diversity in its size, sequence, and structure across eukaryotic clades [4]. This remarkable sequence diversity is a characteristic feature of TR so that simple nucleotide BLAST based approaches have difficulties in determining orthology of TR sequences even between species from closely related genera [26–29]. This level of sequence diversity is also common for many long noncoding RNA genes that are known to be rapidly evolving [30,31].

The TR also contains highly conserved regions that are involved in folding the primary sequence into higher order secondary structures [27, 34] and interacts with TERT for proper telomerase activity [27,35–38]. One of the conserved regions corresponds to the template domain and harbors two sequence motifs that are directly involved in the synthesis of the telomere DNA (see Fig 1 for summary of TERT-TR activity model). The first motif is the annealing sequence (Fig 1 teal sequences) that binds to the single stranded telomere DNA and the second motif is the template sequence (Fig 1 purple sequences) that serves as the template for TERT to synthesize telomeric DNA sequences [39]. Because the TR template sequence dictates the telomeric repeat that are synthesized at chromosome ends, the TR is key for gaining a deep understanding of the evolutionary processes that underlies the eukaryote telomere sequence variation. For instance, past studies in both animals and plants have discovered the lineages that have evolved divergent telomere sequences have also evolved the same changes in the TR template sequence [27–29,34,40]. In addition, evolutionary changes in the TR sequence predict co-evolutionary changes could also occur in the proteins that physically or genetically interact with the TR molecule. Validating this prediction, fungal studies have taken advantage of the powerful yeast genetic system and discovered species with non-canonical telomere repeats have also evolved novel telomere binding proteins (*e.g.,* Rap1 and Taz1) [14].

It is clear sequence changes in the TR underlies the evolution of novel telomere repeat sequences, but what remains enigmatic is how the TR template sequence evolved from its ancestral telomere repeat to a novel repeat sequence. This is a puzzling question, since a mutation within the TR templating sequence will have systematic consequences, where every telomere replication event would be influenced by the mutated template sequence. Studies mutating the TR template sequence have observed abnormal consequences that affects the activity of telomerase, including aberrant telomere extension rates [41,42] and telomerase stuttering that leads to improper sequence synthesized at telomeric ends [43]. The fitness consequence of a mutated template sequence have been rarely tested [44] but given the crucial functions of the telomere, the mutations arising in the TR template sequence are likely to be deleterious for most organisms. This also argues against a hypothesis that genetic drift could lead to the sequence diversity in the telomere. What then is the evolutionary process that generates the genetic variation within the TR template sequence and ultimately the sequence variation in eukaryotic telomeres?

Our evolutionary understanding of TR and telomere evolution largely stems from studies that focused on the telomere sequence variation between species of divergent phylogenetic clades. While these comparisons have been fruitful for surveying telomeric sequence variation across distant taxa, the deep evolutionary scale has stymied the establishment of fine-scale evolutionary processes that are involved in the telomere sequence evolution. In other words, there is a need for studies to examine telomere evolution in closely related species (preferably from the same genus) that have diverged in their telomere sequence. This approach could “catch evolution in the act” and provide insights into the evolutionary processes that are involved in the transition from one telomere repeat type to another. In this study we investigated the evolutionary basis of telomere sequence variation by studying the telomeres of the genus *Mimulus* sensu lato [45,46]. *Mimulus* is a popular system for conducting ecological and evolutionary biology research and has provided key fundamental insights into the genetics and ecology of adaptation and speciation [47,48]. Our findings offers an evolutionary mechanism to explain the telomere sequence turnover in *Mimulus* and with the recent findings of TR duplication across multiple plant lineages [49], our evolutionary model may explain the sequence turnover that is potentially applicable to all eukaryotic telomeres.

## Result and discussion

### Genome analysis uncovers telomere sequence variation between *Mimulus* species.

This project was originally motivated by an attempt to characterize telomere length variation across *Mimulus* species. In our previous study [50], we used the computational method k-Seek [51,52] to count k-mer repeats in raw un-mapped whole genome sequencing reads, and used the telomere repeat k-mer counts as a highly accurate method for approximating the amount of telomere repeats within an individual plant. We used k-Seek to analyze published whole-genome re-sequencing data of *Mimulus* species from three different sections (*i.e.,* morphological groupings): *M. aurantiacus* from section *Diplacus* [53], *M. guttatus* from section *Simiolus* [54], and *M. verbenaceus* from section *Erythranthe* [55]. More recent phylogenetic analyses indicate polyphyly of the genus *Mimulus* [56] and taxonomic revision has split these focal species into separate genera, *Diplacus* and *Erythranthe* [45]. However, following other recent work in the Phrymaceae, we have elected to retain the use of the name *Mimulus* for its historic significance and recognizability (see [46]).

**Fig 2:**
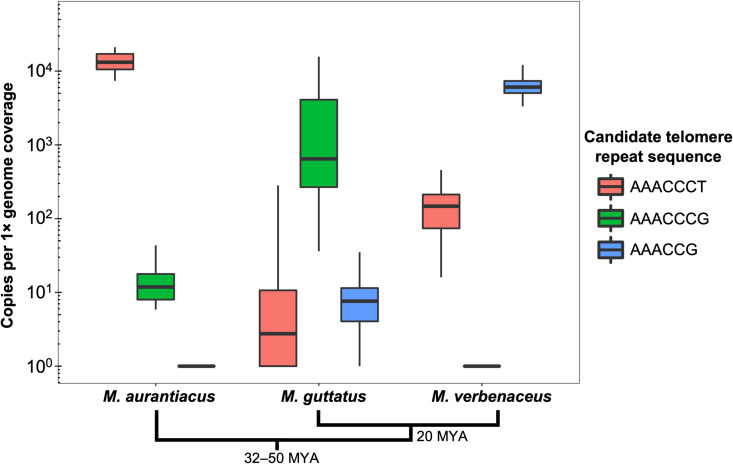
K-mer based genome sequencing analysis identifies changes in the candidate telomere sequence repeat for three *Mimulus* species. For each species only the abundance of the top three k-mers that resemble a telomere sequence are shown on a log10 scale. Phylogenetic relationships between the three species are shown below with their divergence time [57]. Within a species the most abundant k-mer is hypothesized as the species telomere sequence, while the other two k-mers are likely to be low abundance interstitial repeats. Note the observed variability in k-mer repeats represents both natural variation and potential measurement or sequencing errors.

We analyzed *Mimulus* species with population genomic data to quantify the natural variation in k-mer counts (normalized by genome coverage) across multiple genotypes for each species (n = 43 for *M. aurantiacus*, n = 228 for *M. guttatus*, and n = 54 for *M. verbenaceus*). Initially, we did not expect there to be any sequence variation in the telomeres between *Mimulus* species, hence attempted to use the k-mer abundance as an approximation for quantifying telomere length variation. Note for *M. aurantiacus*, the population genomic data are from six closely related subspecies that we collectively refer to as *M. aurantiacus* only for the k-mer analysis for convenience, and importantly our k-mer results do not depend on the underlying subspecies classification. Results showed for all three species, most large sized k-mers (k > 3) had relatively low abundance (~100 copies per 1 × genome coverage), indicating the genome-wide tandem repeat variation was largely driven by small sized 1-, 2-, or 3-mers (S1 Fig). But in *M. aurantiacus* (Fig 2), the k-mer AAACCCT had substantial abundance for a large sized k-mer (~13,190 copies per 1 × genome coverage) and the sequence corresponded to the classical *Arabidopsis*-type telomere repeat sequence (note AAACCCT is an offset of the reverse complement of TTTAGGG [*i.e.,* CCCTAAA] followed by tandem repetition).

The k-Seek approach cannot discriminate the genomic locations the k-mer repeats originated from. Since there are interstitial telomeric sequences that are found outside of chromosome ends [58], we took an independent dataset to check whether the AAACCCT k-mer is terminally located and corresponded to the telomere repeat sequence. We analyzed the *M. aurantiacus* chromosome level reference genome assembly that was sequenced from the subspecies *puniceus* [53], and focused on the sequences at the ends of assembled chromosomes to identify the telomere repeat motif. The chromosome ends were abundant for the k-mer AAACCCT (S2 Fig) confirming that *M. aurantiacus* ssp. *puniceus* harbors the canonical *Arabidopsis*-type telomere sequence.

The genome sequencing k-mer analysis for *M. guttatus* and *M. verbenaceus*, on the other hand, did not detect AAACCCT as the most abundant 7-mer repeat (Fig 2). In fact AAACCCT was almost absent in *M. guttatus* while it had a relatively low abundance in *M. verbenaceus*. This suggested the telomere sequence motif had changed in *M. guttatus* and *M. verbenaceus*. To identify the alternative telomere motif we searched for k-mers that were similar to the *Arabidopsis*-type telomere sequence and had high abundance in the raw genome sequencing data. In *M. guttatus* the k-mer AAACCCG 7-mer had the highest abundance (~645 copies per 1 × genome coverage), and this k-mer was largely absent in *M. aurantiacus* and *M. verbenaceus*. Meanwhile in *M. verbenaceus* there was no highly abundant 7-mer sequence but the 6-mer AAACCG was particularly abundant (~6,070 copies per 1 × genome coverage). Furthermore this k-mer was largely absent in *M. aurantiacus* and *M. guttatus*.

To investigate if the two k-mers AAACCCG and AAACCG corresponded to the telomere repeat we analyzed the chromosome-level reference genome assemblies for *M. guttatus* and *M. verbenaceus*. We focused on the sequences at the chromosome ends and discovered *M. guttatus* assemblies were enriched for the AAACCCG repeat, while *M. verbenaceus* assemblies were enriched for the AAACCG repeat (S2 Fig). These data suggested the abundant k-mers that resemble telomere-like repeats from the *M. guttatus* and *M. verbenaceus* raw genome sequencing data (Fig 2) likely correspond to the true telomere sequences in these species.

### Annotating the telomerase RNA gene in the *Mimulus* species with reference genomes

Our k-mer analysis suggested the telomere sequence had changed at least twice in the *Mimulus* genus (*i.e.,* AAACCCT, AAACCCG, AAACCG). To verify this observation, we studied the evolution of the *Mimulus* TR gene. The template sequence within the TR determines the telomere DNA sequence (Fig 1), hence by investigating the evolution of the TR gene we aimed to gain a deeper understanding of the telomere sequence evolution in the *Mimulus* genus.

We first analyzed the *Mimulus* species with reference genomes (*M. aurantiacus* ssp. *puniceus*, *M. cardinalis*, *M. guttatus*, *M. lewisii, M. parishii*, and *M. verbenaceus*) to identify the TR gene. *Mimulus cardinalis*, *M. lewisii,* and *M. parishii* are species that were not analyzed in our k-mer analysis and belongs to the *Erythranthe* section, which includes *M. verbenaceus* that was included in our k-mer analysis (Fig 2). For each reference genome, we used a position weight matrix [59] and secondary structure-based algorithm [60] to search and annotate the TR gene locations (see S1 Table for genome coordinates). The size of the identified TR genes ranged between 278 bp to 317 bp, which are similar in size to other TRs found across the plant kingdom (ranging from 234–390 bp) [27,28]. During the TR annotation process we also obtained an intriguing result where the reference genomes from *M. aurantiacus* ssp. *puniceus*, *M. cardinalis*, and *M. lewisii* had evidence of two seperate TR gene paralogs. This intriguing discovery and the evolution of these TR duplicates will be further discussed in a later section.

To examine the potential functionality of the annotated TRs we extracted 100 bp upstream of the predicted start site of the TR transcript to search for the presence of conserved regulatory elements. Previous studies have found the upstream genetic regions of land plant TRs contained a highly conserved type III promoter motif called the Upstream Sequence Element (USE) and a TATA box motif [28,61]. All of our candidate TRs, including the duplicates, had the canonical USE motif TCCCACAT within the upstream region (S3 Fig). This sequence was identical to the USE motif found in all surveyed Lamiales species [28]. One exception was *M. cardinalis* where one of its TR duplicates had a single nucleotide substitution in the conserved USE motif (TCCCAC**G**T; the nucleotide difference is bolded) and we verified this nucleotide change with Sanger sequencing. For all candidate TR sequences we also discovered the TATA box 28–30 bp downstream from the USE motif (S3 Fig). In sum, the presence of highly conserved transcription initiation sequences indicated our annotated TRs were likely to be expressed.

### Identifying the telomerase RNA through total RNA transcriptomes of multiple *Mimulus* species

**Fig 3:**
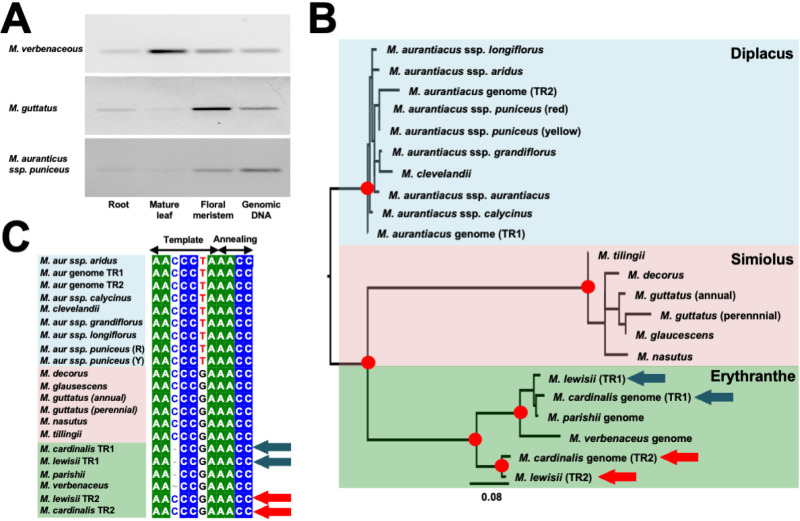
Molecular evolutionary analysis of the *Mimulus* Telomerase RNA (TR) gene. (A) RT-PCR results amplifying TR transcripts on cDNA generated from RNA extractions of three tissues (root, mature leaf, and floral meristem). PCR on genomic DNA is shown as a positive control. (B) Phylogeny of the entire TR sequences assembled from 18 *Mimulus* species/subspecies. Sequences were obtained from total RNA transcriptome sequencing or by annotating the TR sequences from reference genomes (*i.e., M. aurantiacus* ssp. *puniceus*, *M. cardinalis*, *M. parishii*, and *M. verbenaceus*). Nodes with bootstrap support >95% are indicated with a red circle. Phylogenetic groups are colored according to the three *Mimulus* sections. Arrows point to the *M. cardinalis* and *M. lewisii* TR duplication. Blue arrows indicate TR1 and the red arrows indicate TR2. (C) Alignment of the TR templating region (see Fig 1 for the involvement of templating sequence in telomerase complex during telomere DNA synthesis). Sequences are highlighted according to the color scheme of (B). Based on the templating sequence, the *Diplacus* section will synthesize a TTTAGGG telomere repeat and the *Simiolus* section a TTTCGGG telomere repeat. In the *Erythranthe* section, all species will synthesize a TTTCGG telomere repeat. However, since in addition to TR1, *M. cardinalis* and *M. lewisii* have TR2 with a TTTCGGG templating sequence both species could have a potentially sequence heterogeneous telomere sequence (but see Fig 4 for results on the effects of *M. cardinalis* and *M. lewisii* TR duplication on telomere sequence).

We aimed to obtain the TR sequence from multiple *Mimulus* species through *de novo* transcriptomics and assemble the TR transcript from the total RNA pool. We chose the three divergent *Mimulus* species (*M. aurantiacus* ssp. *puniceus*, *M. guttatus*, and *M. verbenaceus*) and synthesized cDNA from total RNA extracted from mature leaf, root, and floral meristem tissues and conducted a RT-PCR analysis. Prior studies have discovered high expression of the TR in plant tissues with actively dividing cells [27,61] and we also detected expression of the TR gene in the floral meristem for all three *Mimulus* species (Fig 3A). We observed no PCR bands when raw total RNA was used for the RT-PCR experiment (S4 Fig), indicating the RT-PCR positive bands did not originate from genomic DNA contamination. There was also some evidence of TR expression in other tissues that are largely composed of differentiated cells (root and mature leaf).

Based on the RT-PCR results we targeted the floral meristem for total RNA transcriptome sequencing with a goal to assemble the TR sequence in multiple *Mimulus* species. We examined the floral meristem transcriptomes from 16 *Mimulus* species/subspecies and these include 8 (sub)species from the *Diplacus* section (*M. aurantiacus* ssp. *aridus*, *M. aurantiacus* ssp. *aurantiacus*, *M. aurantiacus* ssp. *calycinus*, *M. clevelandii*, *M. aurantiacus* ssp. *grandiflorus*, *M. aurantiacus* ssp. *longiflorus*, red flower ecotype *M. aurantiacus* ssp. *puniceus*, and yellow flower ecotype *aurantiacus* ssp. *M. puniceus*), 7 (sub)species from the *Simiolus* section (*M. decorus*, *M. glaucescens*, coastal perennial *M. guttatus*, inland perennial *M. guttatus*, inland annual *M. guttatus*, *M. nasutus,* and *M. tilingii*), and 1 species from the *Erythranthe* section (*M. lewisii*). We also took the TR sequences annotated from the reference genome (listed in S1 Table) for a combined total of 19 (sub)species (see S2 Table for list of analyzed species) TR sequences available for downstream analysis.

After quality control trimmed sequencing reads, we used the trinity pipeline [62] to conduct *de novo* transcriptome assembly. The contig N50 ranged from 617–2619 bp and the number of assembled transcripts ranged from 117,776–266,437 (S3 Table). BUSCO scores were calculated and ranged from 26.4–70.4%. These low BUSCO scores may be related to our total RNA based sequencing strategy. We depleted rRNA but omitted a polyadenylated transcript selection step to enrich noncoding RNA sequences such as the TR, but this does not control for the vastly over abundant organellar (chloroplast and mitochondria) transcripts that are not polyadenylated [63]. Consequently nuclear gene transcripts are likely to be under sampled, potentially resulting in the suboptimal assembly for coding sequences. Despite this limitation, we took the *de novo* transcriptome assembly and using the TR gene annotations from the reference genome assemblies, a BLAST-based nucleotide similarity analysis was able to identify the TR orthologs from 15 of the sequenced transcriptomes (no TR was successfully identified for the inland perennial *M. guttatus*). In the end, we determined the TR sequence from 15 *Mimulus* species from the transcriptome data and additional TR sequences from 3 *Mimulus* species annotated from the genome assemblies (*M. cardinalis*, *M. parishii*, and *M. verbenaceus*) for a total of 18 unique *Mimulus* species.

### The telomerase RNA gene has duplicated in several *Mimiulus* species.

The *Mimulus* TR sequences were aligned to each other with MUSCLE [64] and the multi-sequence alignment was used to build a maximum-likelihood based phylogenetic tree to infer the evolutionary relationships (Fig 3B). The tree had high bootstrap support on internal nodes that grouped the species by *Mimulus* section (>99% after 1,000 bootstrap replicates) and the internal branch lengths between sections were deep. This phylogenetic evidence indicated there were substantial nucleotide differences between the TR sequences for species from different sections. This was also visually apparent in the multi-sequence alignment of the TR gene (S5 Fig), which displayed several insertions and deletions (indels) of variable sizes between *Mimulus* sections.

During the genome annotation and transcriptome-based analysis of the TR gene, we discovered the TR gene was duplicated in several *Mimulus* species (*M. aurantiacus* ssp. *puniceus*, *M. cardinalis*, and *M. lewisii*). We then focused our analysis on the *Mimulus* TR paralogs to understand the evolutionary origin of the duplication event.

In *M. aurantiacus* ssp. *puniceus*, the reference genome harbored two TR gene copies, but our *de novo* assembly from each *Diplacus* species showed evidence of only a single TR gene. We randomly designated the two TR copies from the *M. aurantiacus* ssp. *puniceus* reference genome as TR1 and TR2. Across the 244 aligned basepairs between *M. aurantiacus* ssp. *puniceus* TR1 and TR2 sequences, there were 11 nucleotide differences and a 50 bp insertion found only in TR2 (S5 Fig). We investigated the phylogenetic relationship between *M. aurantiacus* ssp. *puniceus* TR1 and TR2 from the reference genome, and the rest of the *Diplacus* section TR, but the bootstrap supports were too low to make any confident evolutionary inferences (Fig 3B). This suggested a recent evolutionary origin for the likely TR duplication in *M. aurantiacus* ssp. *puniceus*. In addition, our RT-PCR results (Fig 3A) detected the presence of only a single band, and given that the *M. aurantiacus* ssp. *puniceus* TR1 and TR2 genes from the reference genome have a 50 bp difference in size, if both genes were expressed we would expect to see two bands. However, the presence of a single TR band suggested that only one of the TR paralogs is likely being expressed.

*M. cardinalis* and *M. lewisii* were another pair of species where their reference genome assembly had two TR gene annotations. For *M. lewisii* we conducted total RNA transcriptomics analysis and we were able to *de novo* assemble both TR genes, indicating the two TR paralogs were expressed in the *M. lewisii* floral meristem. There was strong phylogenetic support (>95% after 1,000 bootstrap replicates) that grouped TR by paralog and not by species. We designated the TR paralog that grouped with *M. parishii*, and *M. verbenaceus* as TR1, while the duplicate only found in *M. cardinalis* and *M. lewisii* as TR2. Since TR1 and its orthologs were found in all four *Erythranthe* species, the TR1 paralog was hypothesized as the ancestral copy (see later analysis with phylogenetic outgroups that further indicates TR1 is the ancestral paralog) while TR2 was proposed to be the recently derived copy. The branch lengths that separated TR1 and TR2 were deep. Between *M. lewisii* TR1 and TR2 there were 241 basepairs aligned with 31 nucleotide differences, while between *M. cardinalis* TR1 and TR2 there were 251 basepairs aligned with 33 nucleotide differences. The divergence time between TR1 and TR2 was estimated to be ~ 9.3 million years ago, which is older than the species divergence time between *M. cardinalis* and *M. lewisii* (5.5 million years ago) [57].

### The *Mimulus* family telomere sequence evolution

The functional domains within the TR gene (*i.e.,* conserved regions) [27] were largely conserved across the *Mimulus* TR multisequence alignment (S5 Fig), and we then focused our analysis on the TR template domain as it determines the telomere sequence (Fig 1). Within the template domain, the annealing sequence was identical for all *Mimulus* species and it corresponded to the nucleotides AACC. Given that the annealing sequence is crucial for the proper template shift and double-stranded binding between the telomere DNA and TR RNA sequence, it was expected that the annealing sequence would be conserved. But the templating sequence harbored nucleotide differences, specifically between *Mimulus* species from different sections. We discovered all *Diplacus* section species had the templating sequence AACCCTA, all *Simiolus* section species had the templating sequence AACCCGA, and in the *Erythranthe* section the species with the TR1 orthologs had the templating sequence AACCGA (Fig 3C).

In summary, our results indicated the *Mimulus* genus had at least two mutational changes in the telomere sequence motif. Species of the same section had identical telomere sequence motifs, but between species of different sections there was variation in the telomere sequence motif suggesting the evolutionary changes occurred in the common ancestors of each section. Based on these evolutionary changes we propose a single-step mutational model of telomere sequence evolution to explain the genetic variation within the *Mimulus* telomere. The telomere sequence motif AAACCCT is the most commonly observed sequence in plants, hence it is likely to be the ancestral telomere sequence in *Mimulus* and is thus present in the *Diplacus* section species. A single nucleotide change then converted the ancestral sequence to AAACCCG in the common ancestor of the *Simiolus* and *Erythranthe* section. Subsequently in the *Erythranthe* section, a single nucleotide deletion resulted in the formation of AAACCG telomere sequence. A similar evolutionary model has also been proposed in fungi (*i.e.,* termed as the step-by-step model), where progressive changes from an ancestral telomere sequence resulted in the diversity of yeast telomeric repeats [14]. Importantly, this scenario suggests a common evolutionary mechanism or constraint may underlie the telomere sequence variation across the telomeres of all eukaryotes. The model also predicts as the telomere sequence diverges, the telomere binding proteins would display complementary evolutionary changes, as was proposed earlier [65]. Further detailed analysis of this coevolutionary signatures in the telomere protein-DNA complexes would be an intriguing future study.

### The *M. lewisii* TR duplication results in a sequence heterogenous telomere

We next examined the template domain in *Mimulus* species with TR duplications. If the templating sequence between the TR paralogs exhibits natural genetic variation, this could result in the species carrying a telomere with a mixed repeat motif (*i.e.,* a heterogeneous sequence structure). For the TR sequences from the *M. aurantiacus* ssp. *puniceus* reference genome, both TR1 and TR2 paralogs had the identical templating sequence. But in *M. cardinalis* and *M. lewisii* the two TR paralogs differed in the templating sequence. The TR1 paralog had the templating sequence AACCGA, meanwhile the TR2 paralog had the templating sequence AACCCGA. This observation suggests that *M. cardinalis* and *M. lewisii* might synthesize a telomere with a heterogeneous sequence that comprised of both AAACCG and AAACCCG repeats.

**Fig 4:**
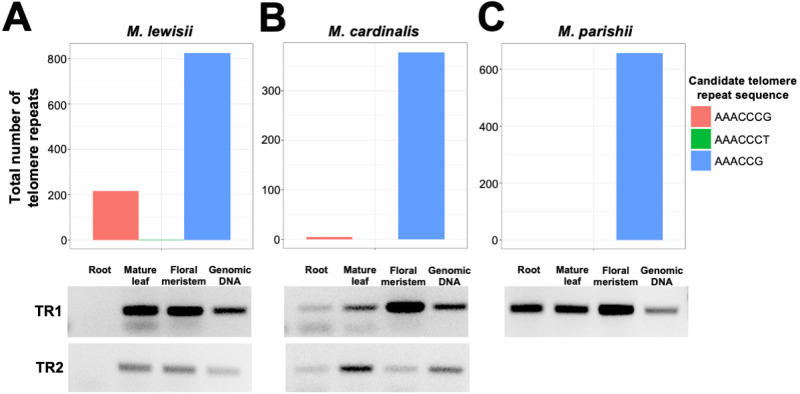
*Erythranthe* section Telomerase RNA (TR) gene duplication and its association with the telomere sequence. Telomere sequence abundance at reference genome assembly chromosome ends (top) and RT-PCR results (bottom) from three tissues (root, mature leaf, and floral meristem) with genomic DNA is shown as a positive control for (A) *M. lewisii*, (B) *M. cardinalis*, and (C) *M. parishii* are shown. See S6 Fig for RT-PCR results from total RNA. Note *M. verbenaceus* is also a species from the *Erythranthe* section and its results have already been shown in Figs 3A and S2, and largely corroborated *M. parishii* results.

We investigated the possible functionality of the *M. cardinalis* and *M. lewisii* TR duplicates by first conducting RT-PCR of the duplicate genes in three different tissues (root, mature leaf, and floral meristem). We first compared the expression profiles of the TR gene in *M. cardinalis* and *M. lewisii*, which have the TR duplications to *M. parishii* and *M. verbenaceus*, which also belong to the same *Erythranthe* section but without the TR duplication. The *M. verbenaceus* TR expression profile and telomere sequence motif analysis were shown in Figs 2, 3, and S2; and *M. parishii* had identical results to *M. verbenaceus* (Figs 4 bottom and S6). In summary, *M. parishii* and *M. verbenaceus* synthesize a single motif telomere sequence (AAACCG)_*n*_ from its single copy TR gene.

For *M. cardinalis* and *M. lewisii*, both TR1 and TR2 were expressed in multiple tissues (Fig 4 bottom), with a particularly relevant expression in the floral meristem, which harbors abundant actively dividing cells. The similar TR gene expression profiles between *M. cardinalis* and *M. lewisii* suggested both species may have a telomere with a heterogeneous sequence structure. We investigated this possibility by quantifying the telomere repeat motifs at chromosome ends of *M. cardinalis* and *M. lewisii* chromosome-level reference genome assemblies, and discovered the two species had contrasting telomere sequences. In *M. cardinalis* only the AAACCG sequence was found at chromosomal ends, meanwhile in *M. lewisii* both AAACCG and AAACCCG sequences were present at terminal chromosomal locations (Fig 4 top). This result for *M.cardinalis* would be consistent with the scenario where TR1 is the only RNA subunit utilized by telomerase, while TR2, though expressed, would not contribute to telomere repeat synthesis and, thus, remain functionally ineffective

### *M. lewisii* has a sequence heterogenous telomere consisting of two telomere repeats.

Given that *M. lewisii* chromosome-level genome assembly suggested the presence of both AAACCG and AAACCCG repeats at chromosomal ends, we conducted a detailed examination of the *M. lewisii* telomere structure by using the Oxford nanopore sequencing platform. This allowed us to extract and analyze the long read sequences that span the entire telomere-subtelomere region. We nanopore sequenced *M. lewisii* and its two closely related species *M. cardinalis* and *M. verbenaceus*, and generated 1.11 Gbp, 0.74 Gbp, and 1.66 Gbp of sequencing data, which comprised of 15,990,860 reads, 10,699,772 reads, and 24,581,417 reads for *M. cardinalis, M. lewisii,* and *M. verbenaceus* respectively (see S4 Table for more sequencing information).

**Fig 5:**
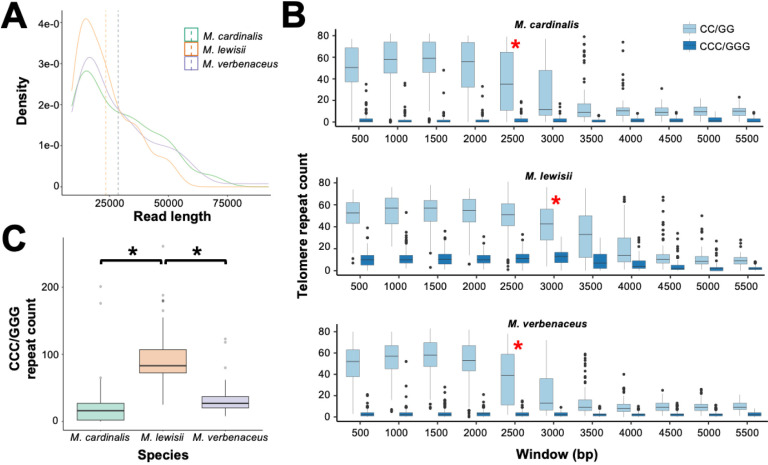
Nanopore long read sequencing of the telomeres in select *Erythranthe* section species. (A) Density plot showing the lengths of sequencing reads that aligned to chromosome ends. Only reads that were longer than 10 kbp were analyzed. Vertical lines are showing the average read lengths which were 28,627 bp for *M. cardinalis*, 28,744 bp for *M. lewisii*, and 23,402 bp for *M. verbenaceus*. (B) 500 bp sliding window analysis of counting CC/GG and CCC/GGG repeats for *M. cardinalis*, *M. lewisii*, and *M. verbenaceus*. The red asterisk indicates the window where there is a significant reduction (Mann-Whitney U test p-value < 0.05) in CC/GG repeat counts. (C) Sum of the CCC/GGG repeat counts up to the 3,000 bp window. Significant differences (Mann-Whitney U test p-value < 0.05) are indicated with a black asterisk.

We matched the sequencing library to its species-specific reference genome, extracted reads that were longer than 10 kbp and aligned them to chromosome ends. For the three species, the total number of nanopore long reads that aligned to the telomere region were 82, 102, and 126 reads with a median read length of 24,995 bp, 22,733 bp, and 20,784 bp for *M. cardinalis, M. lewisii,* and *M. verbenaceus* respectively (Fig 5A). We then conducted a sliding window analysis by starting at the beginning of the telomere sequence (*i.e.,* at the chromosome start or end depending on the arm of the chromosome) and examined the distribution and quantity of the 6-mer AAACCG or 7-mer AAACCCG repeats. Due to the anticipated sequencing errors associated with nanopore reads [66], counting perfectly matched 6-mer or 7-mer telomere repeat would underestimate the number of telomere repeats within the sequencing reads. Instead, a visual analysis of the raw telomere reads indicated that each species had a noticeable difference in the number of CC (and its complement GG) and CCC (and its complement GGG) repeats (S7 Fig). The former likely represented the 6-mer repeat while the latter represented the 7-mer repeats.

A 500 bp window sliding window analysis was conducted for each telomere read and within the window we counted the total number of CC/GG and CCC/GGG repeats (Fig 5B). Results showed for all three species the CC/GG repeat was the most abundant type (~58 counts per window) for all three species, indicating that the 6-mer AAACCG is the predominant telomere repeat. This finding is consistent with our reference genome based analysis results (Fig 4). In addition, the CCC/GGG repeat counts were significantly higher in *M. lewisii* compared to both *M. cardinalis* and *M. verbenaceus* (Fig 5B and 5C; Mann-Whitney U test p-value < 0.05). Noticeably, the CCC/GGG repeat counts were elevated across the windows and up to the candidate telomere-subtelomere boundary region (Fig 5B). This indicated the 6-mer and 7-mer repeats were interspersed throughout the *M. lewisii* telomere.

**Fig 6:**
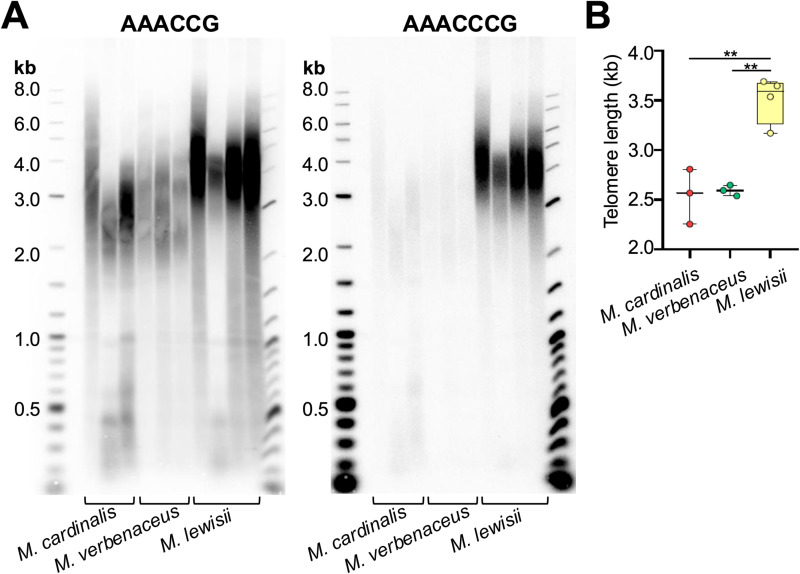
Terminal restriction fragment (TRF) analysis of *M. cardinalis*, *M. verbenaceus,* and *M. lewisii* DNA. (A) TRF Southern blot of DNA samples from individual plants of *M. cardinalis* (n = 3)*, M. verbenaceus* (n = 3) and *M. lewisii* (n = 4) species hybridized to the AAACCG-specific probe (left panel) or re-hybridized after stripping to AAACCCG-specific probe (right panel). Molecular weight DNA markers (in kb) are shown. (B) Telomere length (mean TRF) distributions in ≥3 biological replicates of each genotype from (**A**) are shown in boxplots. Data points represent mean TRF values from individual plants of corresponding species (biological replicates) analyzed with WALTER after hybridization to AAACCG probe. Whiskers indicate maximum to minimum values; boxes represent the lower and upper quartiles (25 and 75%); horizontal lines represent medians of the mean TRF values. ** indicates significant difference p-value < 0.01 after a two-tailed Student’s *t*-*t*est.

We then tested the window where there was a significant reduction in the CC/GG repeat count to detect the regions corresponding to the telomere-subtelomere boundary. In *M. cardinalis* and *M. verbenaceus* it was mapped to the 2,500 bp window, whereas in *M. lewisii* it was considerably larger and corresponded to the window of 3,000 bp (Fig 5B and significant reduction was determined after a Mann-Whitney U test p-value < 0.05). This indicated *M. lewisii* had a potentially 20% longer telomere compared to *M. cardinalis* and *M. verbenaceus*. We validated our telomere length estimates from the genome sequencing data by conducting the Terminal Restriction Fragment (TRF) analysis on *M. cardinalis*, *M. verbenaceus,* and *M. lewisii* DNA. Following restriction digestion of genomic DNA from all 3 species with Tru1I restrictase, we first hybridized the membrane with a 32P-labeled probe to target the AAACCG repeat (Fig 6A, left panel). The probe hybridized to digested DNA samples from all species, confirming our predictions that the AAACCG repeat is universally present on chromosomal ends of all tested *Mimulus* species. Furthermore, quantitative analysis of the TRF signals with the software WALTER [67] indicated that the mean telomere length in *M. lewisii* (3,511 bp) is up to 1,000 bp longer than in both *M. cardinalis* and *M. verbenaceus* (2,543 bp and 2,594 bp, respectively) (Fig 6B, two-tailed Student’s *t*-*t*est p-value < 0.01). This finding further corroborates our telomere length estimates from the nanopore long read sequencing (Fig 5B).

To test for the presence of AAACCCG telomere repeats at the chromosomes of the three *Mimulus* species, we then stripped the AAACCG-specific probe (S8 Fig) and rehybridized the membrane with the AAACCCG-specific probe (Fig 6A, right panel). Remarkably, the AAACCCG-specific probe hybridized equally well to the *M. lewisii* DNA, indicating that chromosomes in this *Mimulus* species harbor a mixture of AAACCG and AAACCCG telomere repeats. Furthermore, the signal for AAACCCG-specific probe was nearly absent from the *M. verbenaceus* DNA, confirming our earlier predictions that telomeres in this species are made up uniquely of AAACCG repeats (Fig 2). Finally, we also detected much weaker AAACCCG-specific signals for the *M. cardinalis* DNA compared to the AAACCG probe in this species. This observation is consistent with the presence of very low amounts of AAACCCG telomere repeats at the *M. cardinalis* chromosomes.

Overall, our TRF analysis with AAACCG and AAACCCG specific probes confirmed that *M. lewisii* is unique among the tested *Mimulus* species in that it uses both types of telomeric templates interchangeably (from two different TR molecules), and that its telomeres are significantly longer than in the other two analyzed *Mimulus* species (two-tailed Student’s *t*-test p-value < 0.01) (Fig 6B). These experimental measurements are very similar to the genome sequencing based estimates we obtained earlier (~3,000 bp for *M. lewisii* versus ~2,500 bp for both *M. cardinalis* and *M. verbenaceus*).

**Fig 7:**
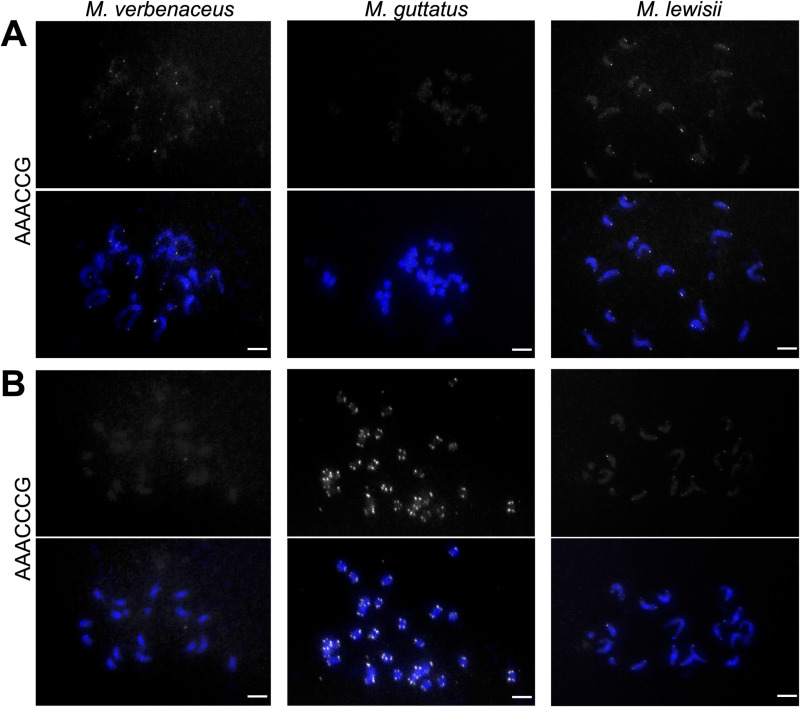
Fluorescence *in situ* hybridization (FISH) on metaphase chromosomes of *M. verbenaceus* (2n = 16), *M. guttatus* (2n = 28), and *M. lewisii* (2n = 16) using (A) AAACCG probe or (B) AAACCCG probe. For each probe the top panel displays the telomere specific FISH signals and the bottom panel displays the DAPI (4’,6-diamidino-2-phenylindole) stained metaphase chromosomes with the telomere specific FISH signals. Bar represents 5 µm.

The nanopore sequencing results and the TRF data were then additionally validated using fluorescence *in situ* hybridization (FISH) experiments to label the telomere repeat sequence of *M. lewisii* chromosomes and compared it to other *Mimulus* species. We labeled the telomeres of *Mimulus* species using a AAACCG probe or a AAACCCG probe and hybridized on metaphase chromosomes (Fig 7). Initially we hybridized the probes on chromosome spreads of *M. verbenaceus* and *M. guttatus*, since both species appears to have a single telomere repeat variant (Fig 2), allowing us to test the specificity of our probes. Results showed that in *M. verbenaceus*, the AAACCG probe hybridized to the chromosome ends but the AAACCCG probe did not show any signal (Fig 7 left panel). Conversely, for *M. guttatus* the AAACCCG probe hybridized to the chromosome ends but the AAACCG probe did not show any signal (Fig 7 middle panel). This indicated the AAACCG probe and AAACCCG probe were specific and hybridized to the corresponding telomeres that matched the probe sequence. In *M. lewisii*, however, both probes were detected at chromosome ends, further confirming that in this *Mimulus* species telomeres consists of a mixture of two different repeat sequences (Fig 7 right panel).

### The telomerase synthesizes AAACCG and AAACCCG telomere repeats in *M. lewisii* but not in *M. cardinalis.*

The heterogeneity of *M. lewisii* telomeres with both AAACCG and AAACCCG telomere repeats suggest the telomerase is able to synthesize both repeat types *in vivo* (Figs 5B, 6 and 7). We conducted RT-qPCR of the TR1 and TR2 transcripts in leaf and meristem tissues and discovered the TR1 gene with the AAACCG template had a significantly higher expression (Mann-Whitney U test p-value < 0.05) compared to the TR2 gene with the AAACCCG template (S9 Fig). We hypothesize that the repeat composition of the *M. lewisii* telomeres is driven by the transcript abundance levels of the TR1 and TR2 genes.

**Fig 8:**
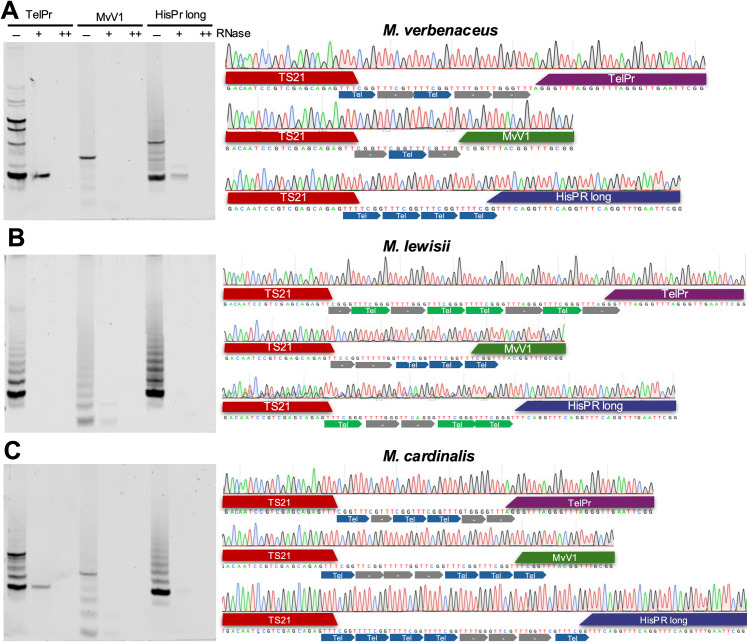
TRAP assays of telomerase activity and sequencing results of cloned TRAP products. Protein extracts from floral meristems of (A) *M. verbenaceus*, (B) M*. lewisii*, and (C) *M. cardinalis* were analyzed. (Left) TRAP products were separated on a polyacrylamide gel and stained for imaging. The (-) lane indicates TRAP products without the addition of RNAse A. The (+) lane indicates TRAP products from an assay including 2 ng of RNAse A and (++) lane indicates TRAP products from an assay including 5 ng of RNAse A. (Right) Representative images of Sanger sequencing of clones generated from positive TRAP assays. Telomere motifs are indicated with colored arrows where AAACCG (*i.e.,* TTTCGG) motif is indicated with blue arrow, AAACCCG (*i.e.,* TTTCGGG) motif is indicated with green arrow, and neither motif is indicated with grey arrow.

We investigated the activity of the telomerase by conducting a Telomeric Repeat Amplification Protocol (TRAP) assay [68]. TRAP is an *in vitro* assay for examining the activity of the telomerase and involves a telomerase-mediated extension of a non-telomeric substrate. We conducted TRAP on *M. lewisii*, *M. cardinalis*, and *M. verbenaceus* protein extracts and sequenced the TRAP products to investigate the repeat sequence synthesized by the telomerase of each species. We used a previously published substrate primer (*i.e.,* TS21) that worked in a variety of plant species and two previously published reverse primers (*i.e.,* TelPr and HisPr_long) that amplified TRAP products with AAACGGG like repeats [49]. We also designed a reverse primer to amplify TRAP products with a AAACCG repeats (*i.e.,* MvV1). All three reverse primers showed positive TRAP products and when the reactions were repeated following RNAse A treatments, the products were not observed indicating the TRAP assay was RNA dependent (left images on Fig 8). The TRAP products were then cloned for Sanger sequencing and only clones that had both primer sequences (*i.e.,* TS21 substrate primer and the reverse primer) and a tandem repeat in between were analyzed. In *M. verbenaceus* we screened a total of 13 clones (TelPr = 3, MvV1 = 5, and HisPr_long = 5) and discovered 17 AAACCG tandem repeats and 1 AAACCCG repeats (see Fig 8 for examples from each reverse primer). This was expected since in *M. verbenaceus* the telomerase is predicted to synthesize the AAACCG repeat sequence. In *M. lewisii* we screened a total of 12 clones (TelPr = 4, MvV1 = 3, and HisPr_long = 5) and discovered 9 AAACCG tandem repeats and 26 AAACCCG repeats. This indicated the *M. lewisii* telomerase was likely using both TR1 and TR2 templates for synthesis and explained the sequence heterogenous *M. lewisii* telomere. In *M. cardinalis* we screened a total of 15 clones (TelPr = 5, MvV1 = 5, HisPr_long = 5) and discovered 28 AAACCG tandem repeats and 3 AAACCCG repeats. Despite both TR1 and TR2 being expressed in *M. cardinalis*, telomerase appears to preferentially synthesized the TTTGCC (*i.e.,* the reverse complement of AAACGG) repeat. The *M. cardinalis* telomerase may have a preference for binding to TR1 or it may preferentially synthesizes the TTTGCC repeat regardless of the TR gene. This preference is also consistent with our TRF data and may also explain why the *M. cardinalis* telomeres largely consist of the AAACCG repeat despite both TR1 and TR2 being expressed in this species.

### TR duplication event was ancient and both copies were retained in *M. lewisii* due to functionality.

It’s possible *M. parishii* and *M. verbenaceus* also had a TR2 gene but it was subsequently lost. This scenario would support the notion that the *M. lewisii* TR duplication was an evolutionarily old event. We conducted a comparative genomic analysis on the TR2 gene region in *M. cardinalis* and *M. lewisii*, and also examined the syntenic region in *M. parishii* and *M. verbenaceus*. Results showed clear synteny across the 4 species in the genomic region surrounding the TR2 gene, which is located on chromosome 7 for all 4 species (S10 Fig). We conducted nucleotide blast analysis using the TR2 sequence as the query and searched for presence of the TR2 in the syntenic regions in *M. parishii* and *M. verbenaceus*. In *M. parishii* we discovered a 36 bp hit (97% identity) and in *M. verbenaceus* a 80 bp hit (88% identity), but no match for the large majority of the TR2 gene was observed. These results indicated *M. parishii* and *M. verbenaceus* did not have a functional TR2 paralog and the sequences that do remain in the genome are potentially pseudogenized gene remnants.

To gain a deeper understanding of the evolutionary history underlying the *M. lewisii* TR duplication, we analyzed the draft genome assemblies [69] from two closely related outgroup species *M. bicolor* and *M. primuloides*. Evolutionarily, *M. bicolor* (section *Monimanthe*) is a phylogenetic sister to the *Erythranthe* section, while *M. primuloides* (section *Monantha*) is a third outgroup. The genomes for these two species were generated from short read (150 bp) Illumina sequencing and their assembly is fragmented, but through blast search we were able to discover the TR gene in both species and together with the *Erythranthe* section TR sequences we reconstructed a phylogenetic tree (S11 Fig). We also used all *Mimulus* TR sequence dataset from Fig 3B to build a phylogenetic tree and the *M. bicolor* and *M. primuloides* formed a monophyletic group with the *Erythranthe* section (S12 Fig). *M. bicolor* had three TR gene copies and one paralog grouped with the TR1 paralogs while the other two grouped with TR2 paralogs. In the outgroup *M. primuloides* there was only one TR gene and phylogenetically it clustered with the TR1 paralogs (but with low bootstraps). *M. primuloides* TR had the templating sequence AACCGA suggesting for all three *Erythranthe*, *Monimanthe*, *Monantha* section *Mimulus* species the (AAACCG)_n_ is the ancestral telomere and the TR1 paralog is the ancestral TR sequence. Further, our evidence suggests TR2 paralog arose from an evolutionarily old duplication event, occurring at the common ancestor of the *Erythranthe* and *Monimanthe* section (~8.2 million years ago) [57]. Most gene duplicates become nonfunctional [70], explaining the pseudogenization of the TR2 paralog in *M. cardinalis*, *M. parishii*, and *M. verbenaceus.* But in *M. lewisii* the TR2 paralog is clearly functional and appears to be directly involved in synthesizing the *M. lewisii* telomere. The initial TR duplication event was old and predates the speciation of *M. lewisii*, suggesting that both TR paralogs have been functional in *M. lewisii* since its evolutionary origin.

### The TR duplication arises through transposition and the ancestral copy is eventually lost.

During the analysis of the *M. lewisii* TR gene duplication we noticed the TR paralogs were located at two different chromosomes. In *M. lewisii*, the TR1 gene was located at chromosome 6 while the TR2 gene was located at chromosome 7 (S1 Table). It was intriguing that the two duplicates were located at physically distant genomic positions and we investigated the evolutionary mechanism that resulted in this pattern using synteny analysis.

**Fig 9:**
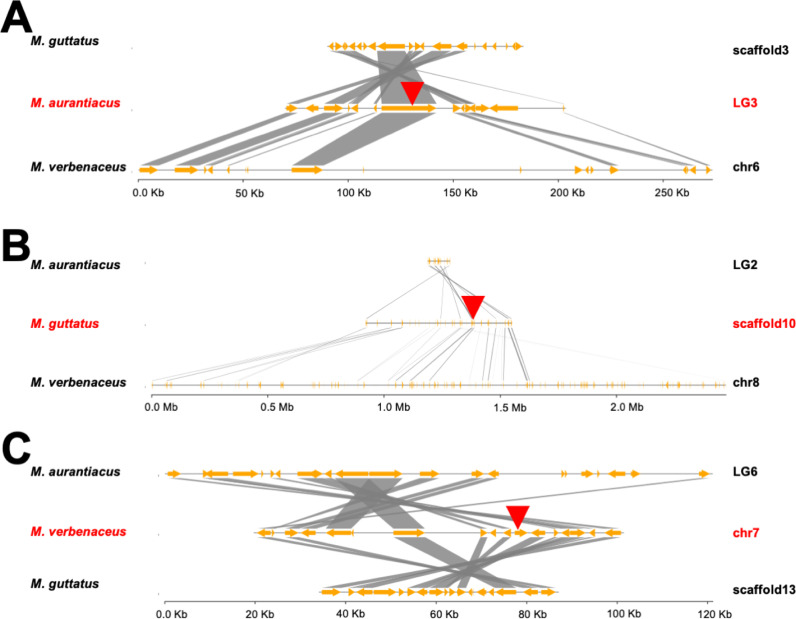
Synteny plot surrounding the Telomerase RNA (TR) gene. Each panel is showing a focal species (A) *M. aurantiacus* ssp. *puniceus*, (B) *M. guttatus*, and (C) *M. verbenaceus*, and its TR chromosomal location (indicated with a red arrow) is plotted in the central position and highlighted with red letters. Orange arrows indicate genes and gray boxes indicate orthology between genes. For each focal species TR gene, we show the five genes upstream and downstream with an orthology. In each plot there is clear synteny between species surrounding a focal species TR gene, but in the non-focal species there is no TR gene and instead it is located at a different chromosome (compare the TR locations in panel A, B, and C).

The genomic region surrounding the *M. lewisii* TR1 gene was highly syntenic with its sister species *M. cardinalis*, *M. parishii*, and *M. verbenaceus* (S10 Fig and S1 Table). We note TR1 in *M. cardinalis* is located at chromosome 7, which seemingly contradicts with the TR1 position of chromosome 6 in *M. lewisii*, but this is due to the TR1 gene located within a natural chromosomal translocation that occurred between chromosome 6 and 7 [71]. We then investigated the synteny of the *M. lewisii* TR1 gene in the evolutionarily divergent *M. guttatus* and *M. aurantiacus* ssp. *puniceus* reference genomes, and discovered those two species did not have a TR gene in the syntenic region (S1 Table). In fact for all three species (*M. aurantiacus*, *M. lewisii*, and *M. guttatus*), the TR gene was located at non-syntenic positions and on different chromosomes (S1 Table and Fig 9). The *M. lewisii* TR2 gene had the same results where syntenic region in the *M. guttatus* and *M. aurantiacus* ssp. *puniceus* reference genomes did not have a TR gene. In summary species in the same section shared TR synteny, but between species of different sections the TR was located at non-syntenic chromosomal positions (S1 Table). A possible scenario to explain these results is if the TR gene is able to duplicate and move to different chromosomes through a transposition-mediated mechanism of gene duplication [72]. We hypothesize this transposition event had occurred in the common ancestor of each *Mimulus* section but then the ancestral TR paralog was lost and the derived TR paralog survived, explaining why species from different *Mimulus* sections do not share TR gene synteny. This turnover of TR gene paralogs ultimately explains the evolutionary basis of telomere sequence variation in *Mimulus*. It is intriguing that each *Mimulus* section with the derived telomere sequence also has the TR gene on a different chromosome, indicating it was potentially the derived TR paralog that was consistently retained. Currently we do not have an evolutionary explanation for this observation, but the retention of the derived TR paralog suggests an unknown selection mechanism may be involved in the process. One hypothesis could be related to genetic conflicts [73,74], as both TR paralogs would interact with telomerase complex for proper function there, which may lead to competition for telomere binding proteins.

The molecular mechanism that led to the transposition of the TR gene is not known, but we speculate transposable elements may have been involved. In six of the nine *Mimulus* species where we annotated the TR gene in the reference genome, there was a transposable element sequence within 2,000 bp of the TR gene annotation and all six of them were a DNA transposon (S5 Table). In plants, DNA transposons have been hypothesized as a major mediator of gene duplication through direct or indirect consequences from the transposition mechanism of the transposon [75]. Thus, we posit the transposition and duplication of the TR gene are potentially mediated through DNA transposons.

## Conclusion

This study aimed to understand the evolutionary basis of telomere sequence variation in *Mimulus*. Specifically, the goal was to understand how the TR sequence evolves and can result in the variable telomere sequence that is commonly observed across the tree of life. To address this aim we investigated the genus *Mimulus*, as our initial genomic analysis discovered between closely related *Mimulus* species the telomere sequence had changed at least twice from the ancestral AAACCCT repeat. The evolutionary basis of the telomere sequence variation was explained by the genetic changes within the templating sequence of TR gene in each species. Importantly we also discovered in specific *Mimulus* species, the TR gene was duplicated and the template sequence had diverged between paralogs. This had resulted in a case like *M. lewisii* where the TR duplication had resulted in its telomere to have a heterogenous mixture of AAACCG and AAACCCG repeats. But in the sister species *M. cardinalis* the duplicated paralog was potentially non-functional and their telomeres consists of a single repeat sequence (*i.e.,* AAACCG). This indicated the functional and evolutionary turnover of the TR paralogs post-duplication has a key role in determining the sequence evolution of the telomere.

**Fig 10:**
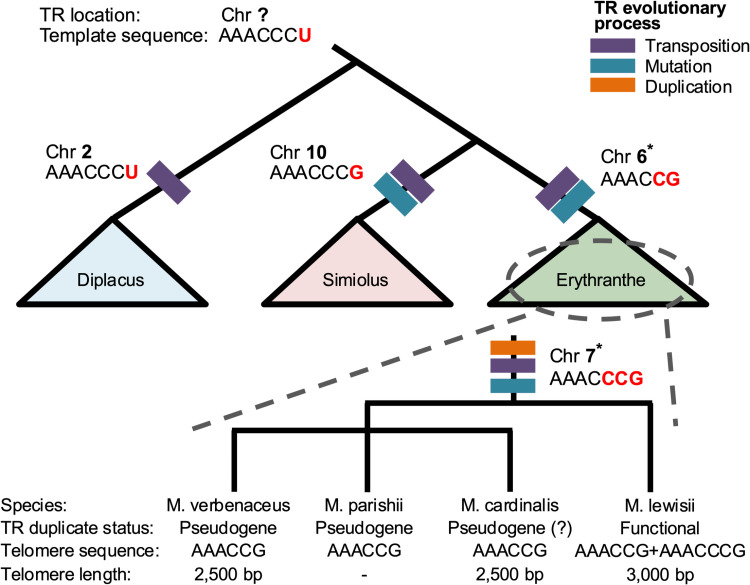
The proposed Telomerase RNA (TR) transposition, duplication, and divergence model to explain the evolutionary changes of eukaryote telomeres. Figure is based on our *Mimulus* results but we argue can be applied across the eukaryotic kingdom. Shown are the phylogenetic relationships among the three *Mimulus* sections (*Diplacus*, *Erythranthe*, and *Simiolus*) that were examined in this study. In the branch leading up to each *Mimulus* section the evolutionary events that shaped the TR gene in the common ancestor of the section are shown with colored bars. In each branch we also show the chromosomal position of the TR gene and the TR templating sequence that ultimately determines the telomere repeat. In the evolutionary model we assume the ancestral telomere sequence in *Mimulus* to be AAACCCT and the evolutionary changes observed within the telomere sequence across the *Mimulus* genus are highlighted in red. We hypothesize the ancestral TR gene was located at an unknown chromosomal position that transposed and inserted into a new chromosomal position during the evolutionary split of each *Mimulus* section. In the *Diplacus* section the ancestral TR transposed to chromosome 2 and there was no mutation in the TR templating sequence, hence all *Diplacus* section species have the ancestral telomere sequence. Within the *Simiolus* and *Erythranthe* section in addition to the section-specific TR transposition events, the TR templating sequence was mutated resulting in species in those sections to have a telomere sequence that is AAACCCG and AAACCG respectively. In the *Erythranthe* section (magnified below) there was TR gene duplication that occurred in the common ancestor of the *Erythranthe* and *Monimanthe* section, resulting in the TR1 and TR2 gene duplicates. We posit TR2 as the recently derived paralog and it originated from a transposition-mediated gene duplication process. The two TR paralogs encode different templating sequences and with *M. lewisii* utilizing both paralogs during telomere synthesis, this results in sequence heterogeneous telomeres consisting of both AAACCG and AAACCCG repeats. While the functional mechanism in *M. lewisii* that leads to evolutionary retention of the TR2 gene is unknown, we hypothesize it could be related to telomere length regulation (telomere length of *M. lewisii* is longer compared to *M. cardinalis* and *M. verbenaceus*). On the other hand, in *M. parishii* and *M. verbenaceus* the TR2 duplicate has pseudogenized, meanwhile in *M. cardinalis* it might be undergoing pseudogenization, hence in all three species the telomere consists predominantly of a single telomere repeat sequence AAACCG. * denote the chromosomal position of the TR based on *M. lewsii* genome coordinates.

From our results we propose the telomerase RNA gene transposition, duplication, and divergence model (see Fig 10 for detail) as the evolutionary mechanism that underlies the telomere sequence variation in *Mimulus* and potentially across various eukaryote lineages. At its core we hypothesize the TR gene can insert into new chromosomal locations through a transposition-mediated duplication mechanism and this has occurred multiple times during the evolution of genomes in the *Mimulus* genus. This duplication opened up the opportunity for sequence mutations to accumulate between the paralogs leading to a heterogeneous telomere sequence in some species. We argue these sequence changes arising between TR paralogs had functional effects and were under selection that resulted in the evolutionarily retention of the TR paralogs. On the other hand, the presence of *Mimulus* species with evidence of pseudogenized TR paralogs suggests non-functional TR duplicates are rapidly lost in evolutionary time. The functional consequence arising from the TR duplication is not known, but one outcome is that the duplication may have influenced the length of the telomere. We discovered *M. lewisii* with the TR duplication had longer telomeres with heterogeneous sequences compared to its sister taxa that have telomeres with a single repeat type (Fig 10). Selection for a long telomere may be the underlying evolutionary driver of TR duplication and sequence evolution. Previously in plants, telomere length variation has been suggested as an evolutionary product of a life-history strategy [32], for instance, rapidly flowering plants have long telomeres in general [50]. Further studies would be necessary to test our hypothesis, but we suggest telomere sequence evolution may have been a product of the life-history evolution and pace-of-life plant strategies.

While our results are based on *Mimulus* telomeres, we argue the duplication and sequence divergence of the TR may be the crucial evolutionary mechanism that ultimately results in the sequence evolution of eukaryotic telomeres. Our discovery also confirms the recent study of Závodník et al. [49] that has reported evidence of the TR duplication in several divergent plant families, indicating the evolution of TR paralogs might be a common plant evolutionary phenomenon. In addition, in the fungi *Candida tropicalis*, some strains have telomeres that are composed of two repeat types differing by a single nucleotide, and it has been proposed the sequence heterogeneity arose from two different telomerase RNA alleles [76]. In summary, testing the generality of our proposed model would require more research in diverse eukaryote lineages, including closely related species of the same genera.

## Materials and methods

### Analyzed reference genomes

All *Mimulus* reference genomes were downloaded from Mimubase (http://mimubase.org/). Specifically, the analyzed genome versions were *M. guttatus* v2.0 [77], *M. aurantiacus* ssp. *puniceus* v1.0 [53], *M. cardinalis* v2.0, *M. lewisii* v2.0, *M. parishii* v2.0, and *M. verbenaceus* v2.0. We note the genome sequences for *M. cardinalis*, *M. lewisii*, *M. parishii*, and *M. verbenaceus* are early access publicly available versions and a manuscript describing the biological properties of those genomes will be published elsewhere.

### k-Seek based telomere sequence analysis.

We downloaded *M. aurantiacus*, *M. guttatus*, and *M. verbenaceus* whole genome resequencing data [53–55] from NCBI SRA with the identifiers PRJNA549183, PRJNA344904, and PRJNA813304. Raw reads were trimmed using BBTools (https://jgi.doe.gov/data-and-tools/bbtools/) bbduk.sh script v39.01 with parameters minlen = 25 qtrim = rl trimq = 10 ktrim = r k = 25 mink = 11 hdist = 1 tpe tbo. Trimmed reads were then analyzed with k-Seek [51,52] to quantify the total copy number of a tandem repeat sequence. K-Seek identifies tandem repeat motifs of up to 20 bp in the raw sequencing reads. It breaks down the sequencing read into smaller fragments (*i.e.,* k-mers) and groups it into sequence identity groups. This allows identification of k-mers and its subsequent quantification by analyzing all sequencing reads.

k-Seek requires a k-mer repeat length to be at least 50 bp in order to avoid over counting small tandem repeats scattered across the genome. To account for the differences in genome coverage between samples we normalized each sample’s k-mer counts by the sample’s median genome coverage. Genome coverage was calculated by first aligning the whole genome resequencing reads to the reference genome of the respective species using bwa-mem v0.7.16a-r1181 [77] and then the per-sample average coverage was calculated using bedtools v2.25.0 [78].

### Reference genome based telomere sequence analysis

All *Mimulus* species have a reference genome that is assembled into chromosomes (*i.e.,* linkage groups), hence if the telomere sequence was assembled it would be at the ends of each chromosome assembly. From each species reference genome we extracted the starting and ending 500 bp of the assembled chromosome sequence using samtools v1.14 [79]. For each extracted sequence we then searched for the telomere repeat sequences AAACCCT, AAACCCG, or AAACCG using bash command grep with option -aob and counted the number of matches. The data was visualized using ggplot2 package [80] in R v4.3.1 (RStudio 2020).

### Plant material

The *Mimulus* species that were used in this study with the growth conditions are mentioned in S2 Table. Total genomic DNA was isolated from the leaves of *M. cardinalis*, *M. guttatus*, *M. aurantiacus* ssp. *puniceus*, *M.parishii, M. verbenaceus*, and *M. lewisii* using the modified CTAB method [81].

### RNA isolation and reverse transcription-polymerase chain reaction (RT-PCR).

The total RNA was extracted from 100 mg of floral meristem, root, and leaves using TRIzol (Invitrogen) according to the manufacturer’s protocol. The RNA was further treated with DNaseI (RNase-free, New England Biolabs) followed by column purification using Monarch Total RNA Miniprep Kit (New England Biolabs). The ProtoScript First Strand cDNA Synthesis Kit (New England Biolabs) was used for the synthesis of cDNA using the manufacturer’s protocol. One µg of RNA was reverse transcribed using random primers.

RT-PCR was performed on the root, leaves, and floral meristem of *M. cardinalis*, *M. guttatus*, *M. aurantiacus* ssp. *puniceus*, *M.parishii, M. verbenaceus*, and *M. lewisii*. Four µl of 10X diluted cDNA was used in a 20 µl reaction mix. Taq DNA Polymerase with Standard Taq Buffer (New England Biolabs) was used for the amplification of TR under conditions as follows: 10 min at 95°C; 30 sec at 95°C; 30 cycles of 30 sec at 95°C, 30 sec at 55°C, 15 sec at 68°C; and final extension at 68°C for 5 min. The primers used for the RT-PCR are listed in the S6 Table. The PCR products were separated using gel electrophoresis at 95 volts for 40 minutes on a 2% agarose gel stained with ethidium bromide.

### RNA-seq library preparation

The total RNA extracted from the floral meristem of species listed in S2 Table were checked for quality using RNA ScreenTape (Agilent Technologies) and Qubit 2.0 fluorometer was used for determining the concentration. From the total RNA the ribosomal RNA (rRNA) were depleted using QIAseq FastSelect–rRNA Plant Kit. Using rRNA depleted RNA samples the RNA-seq libraries were prepared using NEBNext Ultra II Directional RNA Library Prep Kit according to the manufacturer’s protocol. RNA-seq libraries were sequenced by the University of Kansas Genome Sequencing Core on a Illumina NextSeq 2000 platform using P3 Reagents and as 2 × 100 bp paired-end reads.

The total number of raw reads generated for each transcriptome ranged from 102–182 million reads and more than 98% of those reads were recovered after quality control trimming to remove adapters and low quality sequence regions (S3 Table). We then aligned the quality control trimmed reads back to a reference genome to check for the proportion of reads that originated from a genome. Using *M. aurantiacus* ssp. *puniceus* reference genome for *Diplacus* species transcriptomes, *M. guttatus* reference genome for *Simiolus* species transcriptomes, and *M. lewisii* reference genome for the *M. lewisii* transcriptome, we determined there were between 50.0–99.7% of the reads aligned back to a reference genome. The low alignment percentage for some species is likely due to the reference genome being too divergent from the species with RNA-seq reads to be mapped.

### *De novo* transcriptome assembly

From the raw sequencing reads, the quality of reads were analyzed using fastqc v0.11.8 (https://www.bioinformatics.babraham.ac.uk/projects/fastqc/). The reads were subjected to quality control trimming using the bbduk.sh script in the BBMap package v39.01 (https://sourceforge.net/projects/bbmap/). The processed data was used for *de novo* transcriptome assembly using the Trinity pipeline v2.8.5 [62] and following their assembly tutorial (https://github.com/trinityrnaseq/trinityrnaseq/wiki). The *de novo* assembly was runned as paired-end option with the command: Trinity --seqType fq --left “$FQ1” --right “$FQ2” --CPU 40 --max_memory 140G --output “$OUTDIR”. To align the reads back to the reference genome and check for read alignment statistics we used the program Bowtie2 v2.3.5.1 [82]. TrinityStats.pl script from the Trinity pipeline was used to compute transcript contig N50 values. To quantify the completeness of the transcriptome assemblies, the Benchmarking Universal Single-Copy Orthologs (BUSCO) v3.0.2 [83] was executed with the following command: run_BUSCO.py -c 20 -o OUTPUT_NAME --in SEQUENCE_FILE -l eudicotyledons_odb10 -m transcriptome.

### Telomerase RNA gene bioinformatic analysis

Candidate TR gene annotation was conducted using a previously implemented bioinformatics methodology that was used for detecting the TR in various land plant species [27]. We used TR sequences from previously annotated plants to generate a position weight matrix (PWM) to search for the candidate TR sequences within the *Mimulus* reference genomes using fragrep2 [59], and the program Infernal [60] was used to search for candidate TRs using secondary structure and sequence similarity.

To search for the TR sequence within the *de novo* transcriptome assembly we used the TR gene that was annotated from the reference genome using fragrep2 and Infernal. Using the standalone blast+ v2.9.0 [84] the candidate TR gene in the transcriptome assemblies were identified using the following command: blastn -query “$QUERY_FASTA” -db “$REF_DB” -task blastn -outfmt 6. The closely related *Mimulus* species from the same section was used as the query sequence. The TR gene detected from each *Mimulus* species were aligned to each other using MUSCLE [64] with default parameters on MEGA v5.0 [85].

### Annotating conserved and functional regions in the *Mimulus* TR

For species with the reference genome, the 100 bp upstream region of each species TR gene was retrieved for analyzing the conserved regulatory elements. The sequences were aligned to each other using MUSCLE [64] with default parameters on MEGA [85]. We specifically amplified the *M. cardinalis* TR2 upstream region and Sanger sequenced using primers mentioned in S6 Table. In addition, we annotated the functional domains with the *Mimulus* TR sequence using *A. thaliana* TR sequence. Since previous *A. thaliana* study had experimentally validated the secondary structure and functional domains of the TR [27], we aligned the *A. thaliana* TR sequence with the *Mimulus* TR multi-sequence alignment and annotated the *Mimulus* TR functional domains.

### Phylogenetic analysis

The multi-species TR gene alignment was used for the phylogenetic analysis with RAxML v8.2.5 [86] to build a maximum likelihood-based tree. We used a general time-reversible DNA substitution model with gamma-distributed rate variation and bootstrap analysis was conducted with 1000 replicates.

Divergence time between the two TR duplicates was estimated using Nei’s genetic distance (Nei 1972) (*D = 2μT* where *D* is genetic divergence between the gene paralogs, *μ* is the mutation rate per generation, and *T is* number of generations). We used mutation rate estimate from *A. thaliana* (7E-9 base substitutions per site per generation) [87] and assumed 1 generations per year.

### DNA isolation and library preparation for nanopore sequencing

For DNA extraction, leaves of *M. lewisii*, *M. cardinalis*, and *M. verbenaceus* were collected. High Molecular Weight DNA isolation was perfomed using the Wizard HMW DNA Extraction Kit (Promega). Library preparation was performed according to the Ligation Sequencing Kit V14 (SQK-LSK114) protocol from Oxford Nanopore Technologies (ONT). For each library, 1 μg of DNA was used as an input for DNA repair and end preparation for adapter attachment. DNA was treated with NEBNext FFPE DNA Repair Mix (NEB, M6630) and Ultra II End-prep Enzyme Mix (NEB, E7546) followed by purification using AMpure XP beads (AXP) and eluted in 61 μl of nuclease-free water. To attach sequencing adapters to DNA ends, 60 μl of eluted DNA from previous step was ligated with 5 μl of ligation adapter using NEBNext Quick T4 DNA Ligase (NEB, E6056) for 10 mins at room temperature. AXP beads were added and washed twice with Long Fragment Buffer (LFB). The final library was eluted in 15 μl of elution buffer and prior to sequencing DNA amount was quantified using Qubit. The library was kept on ice until ready to load on the MinION device.

### MinION sequencing

The library was sequenced of a MinION Flow Cell (FLO-MIN114 R10.4.1 version) on a Mk1C (Oxford Nanopore Technologies) sequencer. Before sequence run start, a flow cell check was performed to determine the number of active pores available in flow cell. A total of 1000 μl of flow cell priming mix was loaded on priming port and a total of 75 μl of library mixed with Library Beads (LIB) was loaded on the SpotON sample port.

### Data analysis

MinION base-calling was carried out by Guppy v3.6.1 + 249406c (https://nanoporetech.com/software/other/guppy). The reads were aligned back to reference genome using minimap2 v2.25 [88], using following command: “minimap2 -L -t “$CPU” -ax map-ont “$refgenome” “$fastq” > “$FILENAME”.sam. Nanopore reads aligning to reference genome telomere sequence region were extracted with samtools and the reads were analyzed with the grep command to count the telomere sequence repeat.

### Terminal Restriction Fragments (TRF) analysis

Telomere length was measured by the Terminal Restriction Fragments (TRF) analysis [89] with minor modifications. Genomic DNA from individual plants was digested with Tru1l (ThermoFisher Scientific) restriction enzyme, and the digested DNA samples were separated by gel electrophoresis in 0.8% agarose gel at 55V for 18 h in 1X TAE buffer and transferred to a Hybond-N+ nylon membrane (GE Healthcare, Chicago, IL, USA). Digested DNA was hybridized with 32P-labeled CGGTTTCGGTTTCGGTTTCGGTTTCGGT probe for telomeric DNA sequence detection. CGGTTT-specific probe was subsequently stripped from the membrane (by incubating with 0.2N NaOH, 0.1% SDS at 42°C with shaking for 20 min twice), and reprobed with the 5’ end labeled CGGGTTTCGGGTTTCGGGTTTCGGGTTT probe. Radioactive signals were scanned with Amersham Typhoon IP PhosphorImager (Cytiva, Wilmington, DE, USA). Images were visualized with ImageQuantTL v10.2-499 software (Cytiva, Wilmington, DE, USA), and mean telomere length values (mean TRF) for each DNA sample were calculated using the WALTER program [67].

### Real-time quantitative PCR (qPCR)

A quantitative real-time PCR was performed in *M. lewisii* leaves and floral meristem to check the level of expression of the TR paralogs. The qPCR was performed using PowerUp SYBR Green Master Mix (Applied Biosystems) on QuantStudio 3 Real-Time PCR System (Applied Biosystems). Each 10 μl reaction consisted of 5 μl of PowerUp SYBR Green Master Mix (2X), 1 μl of each primer, 2 μl of cDNA, and 1 μl of nuclease free water. The details of primer used in qPCR are mentioned in S6 Table. The reaction setup was as follows: initial activation at 50 °C for 2 min, 95 °C for 2 min followed by 40 cycles of denaturation at 95 °C for 15 s, and annealing/extension for 1 min at 60 °C in 96-well optical reaction plates. In the end dissociation step was performed in 3 steps, first at a ramp rate of 1.6°C/sec at 95 °C for 15 sec, second at 60 °C for 1 min and final at 0.15°C/second at 95 °C for 15 sec. The PCR efficiency was calculated using Data Analysis for Real-Time PCR (DART-PCR) [90].

### Telomerase Repeat Amplification Protocol (TRAP) assay

The shoot apical meristem and floral buds of *M. lewisii*, *M. verbenaceus*, and *M. cardinalis*, were grounded in mortar and pestle using liquid nitrogen for protein isolation. Approximately, 100 mg of crushed tissue was added to 800 μl of Buffer W (50 mM Tris-acetate (pH 7.5), 5 mM MgCl_2_, 100 mM potassium glutamate, 20 mM EGTA, 1.5% (wt/vol) polyvinylpyrrolidone, and 10% glycerol) containing 0.6 mM ribonucleoside vanadyl complex and 1.0 mM DTT. Extracts were incubated on a rotator for 10 minutes at 4°C, and centrifuge at 13,000 rpm for 15 min at 4°C. In the supernatant 350 µL of 50% PEG (6000 MW) was added and were incubated on a rotator for 30 min at 4°C. The products were then centrifuged at 8,000 rpm for 5 min at 4°C and the supernatant was discarded. A 100 μl of Buffer W was added and the pellet was gently resuspend and placed on a rotator for 30 min at 4°C [91]. The extracted protein was stored at -80°C until use. Protein concentration of extracted samples were determined using Bradford’s reagent.

Telomerase activity of extracted protein was tested using TRAP. Briefly, 500 ng of total protein was mixed with 1 µl of 10 µM TS21 (listed in S6 Table), 1X Taq PCR Master Mix (Cat. No. ID: 201445) and incubated at 37 °C for 45 mins. After extension, 1 µl of 10 µM reverse primer was added (listed in S6 Table), followed by PCR step of the TRAP assay (94°C for 15 min, 30 cycles of 94°C for 30 sec, 60°C for 30 sec, 72°C for 60 sec, with a final extension of 72°C for 1 min). For control experiment RNase was added in two different concentration, 40mg (+), and 80 mg (++). PCR products were separated on 10% native polyacrylamide gel in 0.5x TBE buffer and visualized after staining with SYBR Green I Nucleic Acid Gel Stain (Invitrogen, S7567) and visualized using LI-COR Odyssey M [92,93]. Reaction products from TRAP assay were cloned into a pCR2.1-TOPO plasmid using the TOPO TA Cloning Kit (Invitrogen, K450002). Positive clones were sent for Sanger sequencing.

### Fluorescence in situ hybridization (FISH) analysis

Chromosome preparation and FISH procedure were performed following a previous method [94] with minor modifications. Mitotic chromosome spreads were prepared from the root tips of actively growing *Mimulus* plants, which were placed in a nitrous oxide gas chamber for 1.5 h. The root tips were then fixed overnight in a solution of ethanol and glacial acetic acid (3:1) and squashed with a drop of 45% acetic acid. All preparations were stored at -70°C until use.

After carefully removing the coverslips with a double-edged razor blade, the slides were pre-treated with 20 μg/mL pepsin in 10 mM HCl for 3 min at 37°C, followed by immersion in distilled water for 1 min to stop the pepsin reaction. The pre-treated slides were then washed three times with 2x SSC (300 mM Na-citrate, 30 mM NaCl, pH 7.0) for 5 min each. Subsequently, the slides were treated with 4% formaldehyde in 2x SSC for 5 min, dehydrated through a graded ethanol series (70%, 90%, and 100%, 2 min each), and air-dried.

The telomere specific DNA probes that targeted the AAACCCG and AAACCG repeat was developed by PCR in the absence of template DNA [95,96]. The PCR products were purified using QIAquick PCR purification kit (Qiagen) and labeled with digoxigenin-11-dUTP (Roche) using a nick translation reaction. Digoxigenin-labeled probes were detected with a rhodamine-conjugated anti-digoxigenin antibody (Roche). Chromosomes were counterstained with 4′,6-diamidino-2-phenylindole (DAPI) in Vectashield antifade solution (Vector Laboratories). The images were captured using a Zeiss Axioplan 2 microscope equipped with a cooled CCD camera CoolSNAP HQ2 (Photometrics) and AxioVision 4.8 software. The final contrast of the images was adjusted using Adobe Photoshop 2024 software.

### Synteny analysis

Synteny of the TR gene was determined through orthology of the coding sequence surrounding the TR. For each *Mimulus* species with a reference genome we took the chromosomal position of the TR and extracted the gene annotations that were up and downstream of the TR gene. For the focal species and its TR gene, orthology of the surrounding genes were determined through Orthofinder v2.5.5 [97,98] and using gene annotations of *Mimulus* species downloaded from Mimubase (http://mimubase.org/). In order to visualize the synteny, pyGenomeViz was used by following the tutorial (https://github.com/moshi4/pyGenomeViz).

### Supporting information

S1 Table Genome coordinate and sense of the telomerase RNA (TR) gene in species with a reference genome. For each TR gene its syntenic region in other species is also shown. The syntenic region was determined from orthology of the gene directly upstream and downstream of the TR gene. The reported coordinates in the syntenic region are the end position of the upstream gene and start position of the downstream gene. Note because of micro-synteny differences the exact location of a TR and its syntenic position may not completely overlap (*e.g.,* see TR2 gene position in *M. lewisii* reference genome and the syntenic position based on *M. cardinalis* TR2).
(XLSX)

S2 Table The *Mimulus* species that were studied in this research. (XLSX)

S3 Table The *Mimulus* transcriptome *de novo* assembly statistics. (XLSX)

S4 Table The *Mimulus* nanopore sequencing statistics. (XLSX)

S5 Table The transposable element sequence directly adjacent to the TR gene. (XLSX)

S6 Table The primer sequences that were used in this research. (XLSX)

S1 Fig Genome-wide tandem repeat profiles for species (A), (B), and (C). Top 25 most abundant k-mers are shown with the k-mers ordered alphabetically then by size.
(PDF)

S2 Fig Telomere repeat sequence counts at ends of reference genome assembly. (PDF)

S3 Fig Upstream 100 bp of sequence alignments of candidate TR genes from reference genomes. (PDF)

S4 Fig RT-PCR results amplifying TR transcripts on cDNA generated from RNA extractions of three tissues (root, mature leaf, and floral meristem) and the raw RNA as negative control. Genomic DNA is shown as a positive control.
(PDF)

S5 Fig Alignment of the entire *Mimulus* TR gene. The functional domains CR1-CR5 are indicated above the alignment.
(PDF)

S6 Fig RT-PCR results amplifying TR transcripts on cDNA generated from RNA extractions of three tissues (root, mature leaf, and floral meristem) and the raw RNA as negative control. Genomic DNA is shown as a positive control.
(PDF)

S7 Fig Nanopore sequencing read from chromosome 7 telomere region. For each species a random nanopore reads was chosen to display the DNA sequence corresponding to the telomere region. GG nucleotides are highlighted in green while GGG nucleotides are highlighted in red. TTTC sequence is highlighted in yellow.
(PDF)

S8 Fig Southern blot image after stripping away the AAACCG-specific probe. Molecular weight DNA markers (in kb) are shown.
(PDF)

S9 Fig RT-qPCR results amplifying the transcripts of TR1 and TR2 gene. Experiment was conducted with 3 biological replicates.
(PDF)

S10 Fig Synteny plot of the *M. lewisii* and *M. cardinalis* TR1 and TR2 region in *M. parishii* and *M. verbenaceus.* The TR chromosomal location is indicated with a red arrow. Orange arrows indicate genes and gray boxes indicate orthology between genes. We show the five genes upstream and downstream with orthology.
(PDF)

S11 Fig Phylogeny of the TR gene from species in the *Erythranthe* section (*M. cardinalis, M. lewisii, M. parishii, and M. verbenaceus*) and outgroup *M. bicolor* and *M. primuloides.* The TR gene phylogeny shows sequences grouping by paralog. The templating sequence within the TR paralog is shown on the right. Internal nodes represent bootstrap support after 1,000 replicates.
(PDF)

S12 Fig Phylogeny of the entire TR sequences assembled from 18 *Mimulus* species/subspecies total RNA transcriptome (Fig 3B) and *M. bicolor* and *M. primuloides* TR sequences. Nodes with bootstrap support >95% are indicated with a red circle.
(PDF)
