# Supplementary figures and images for: Monkeyflower (Mimulus) uncovers the evolutionary basis of the eukaryote telomere sequence variation
Source: PLoS Genet. 2025 Jun 16;21(6):e1011738. doi: 10.1371/journal.pgen.1011738 (PMC12169523; doi:10.1371/journal.pgen.1011738)

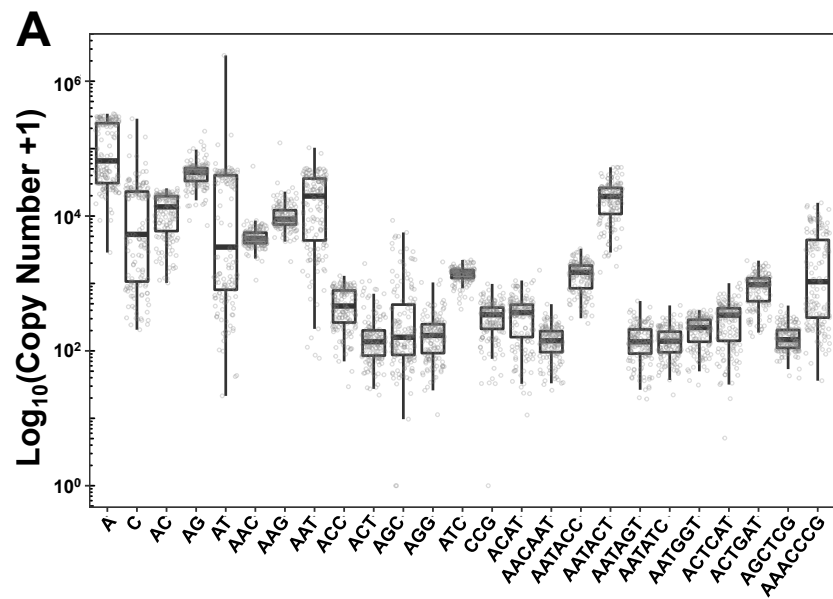

*M. guttatus*

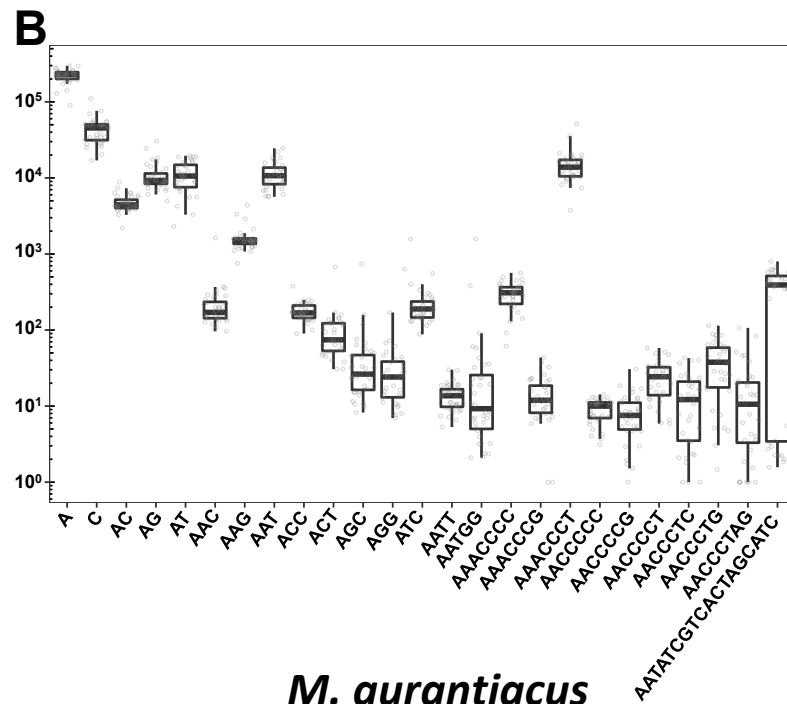

*M. aurantiacus*

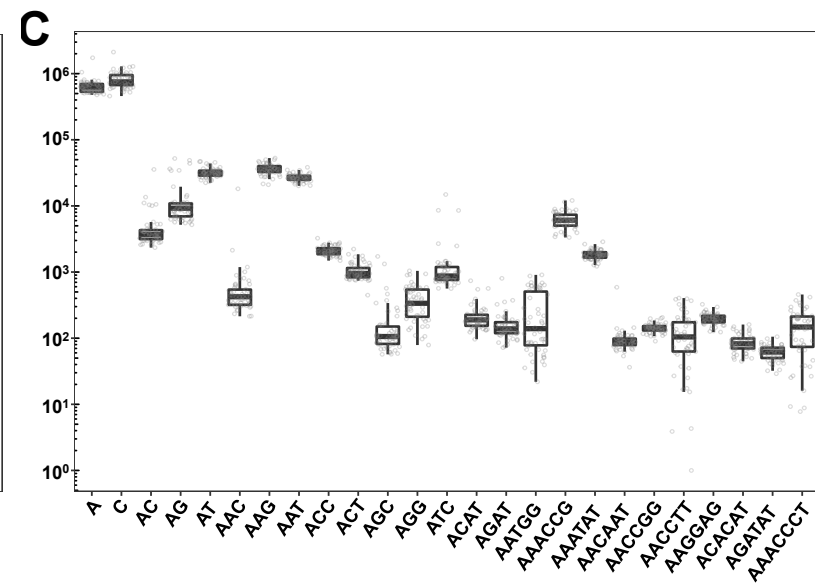

*M. verbenaceus*

Supplement: S1 Fig — Top 25 most abundant k-mers are shown with the k-mers ordered alphabetically then by size. (PDF) [file pgen.1011738.s007.pdf]

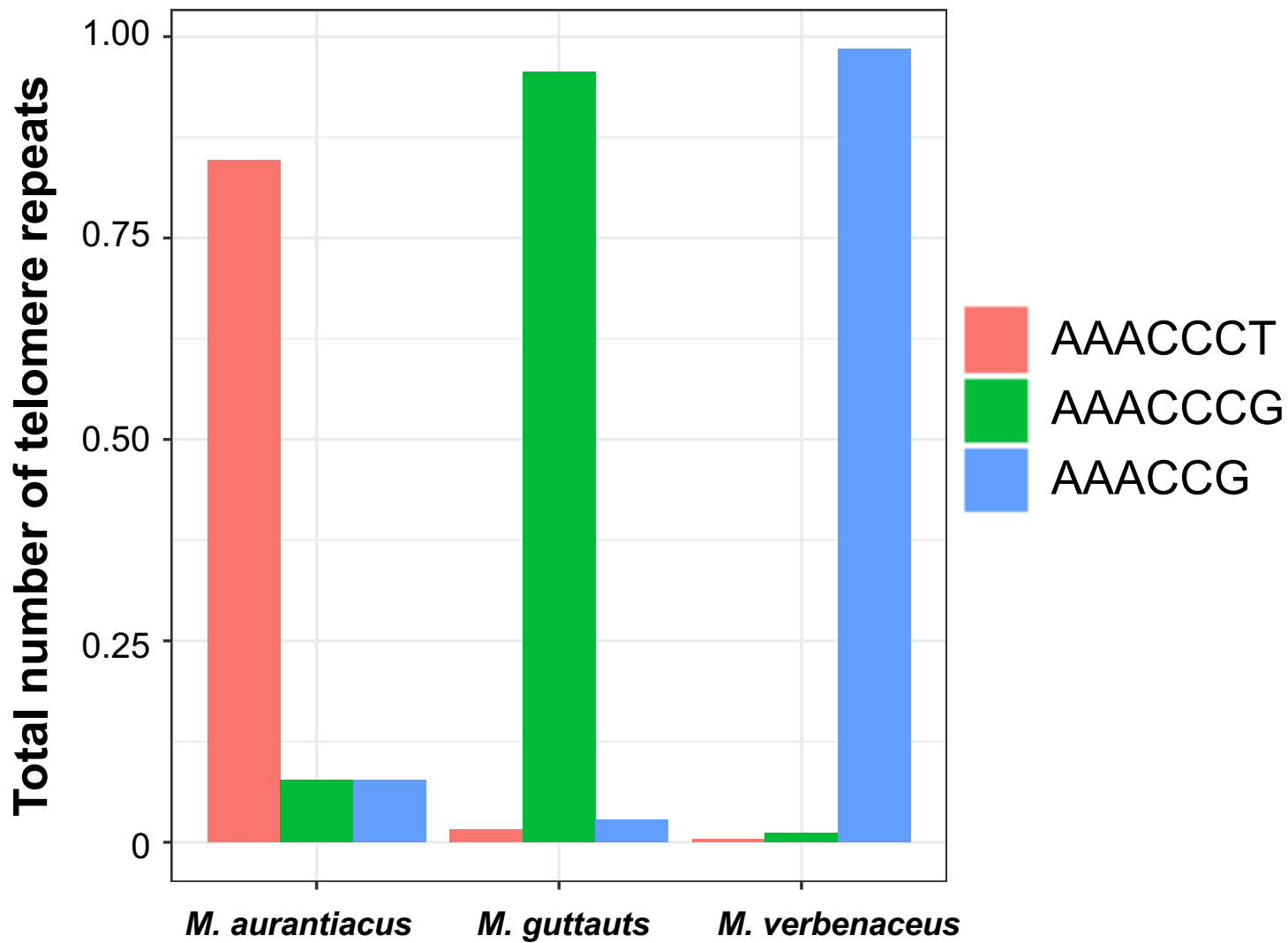

Supplement: S2 Fig — (PDF) [file pgen.1011738.s008.pdf]

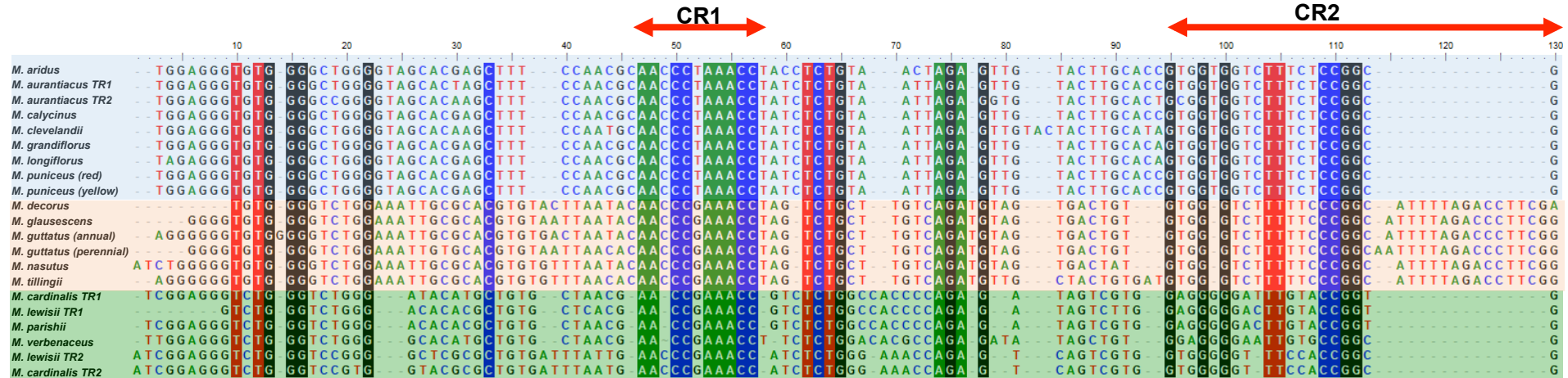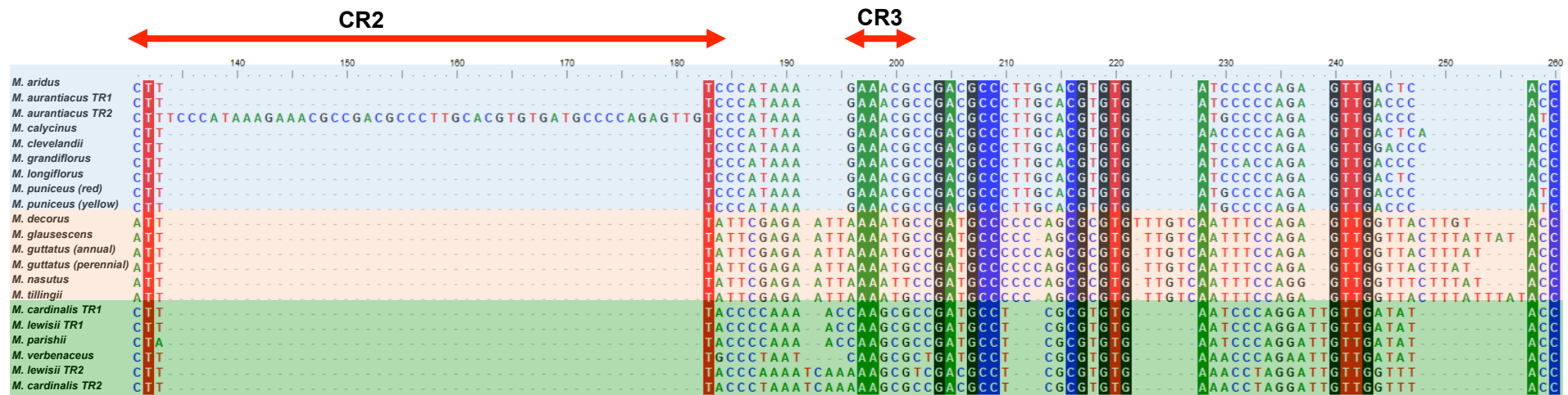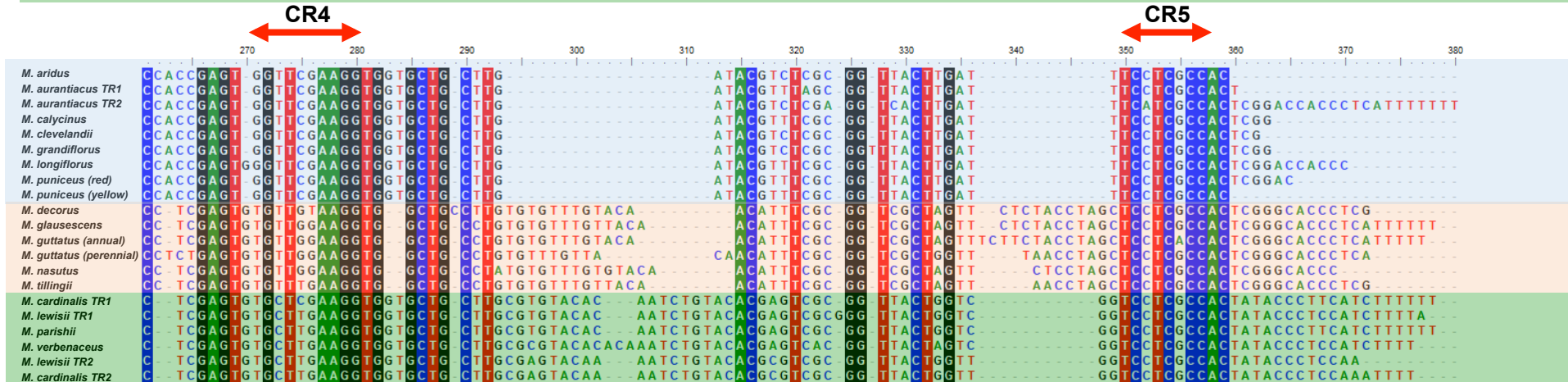

Supplement: S5 Fig — The functional domains CR1-CR5 are indicated above the alignment. (PDF) [file pgen.1011738.s011.pdf]

*M. cardinalis*  
*M. verbenaceus*  
*M. lewisii*

8.0 kb

6.0 kb

4.0 kb

3.0 kb

2.0 kb

1.0 kb

0.5 kb

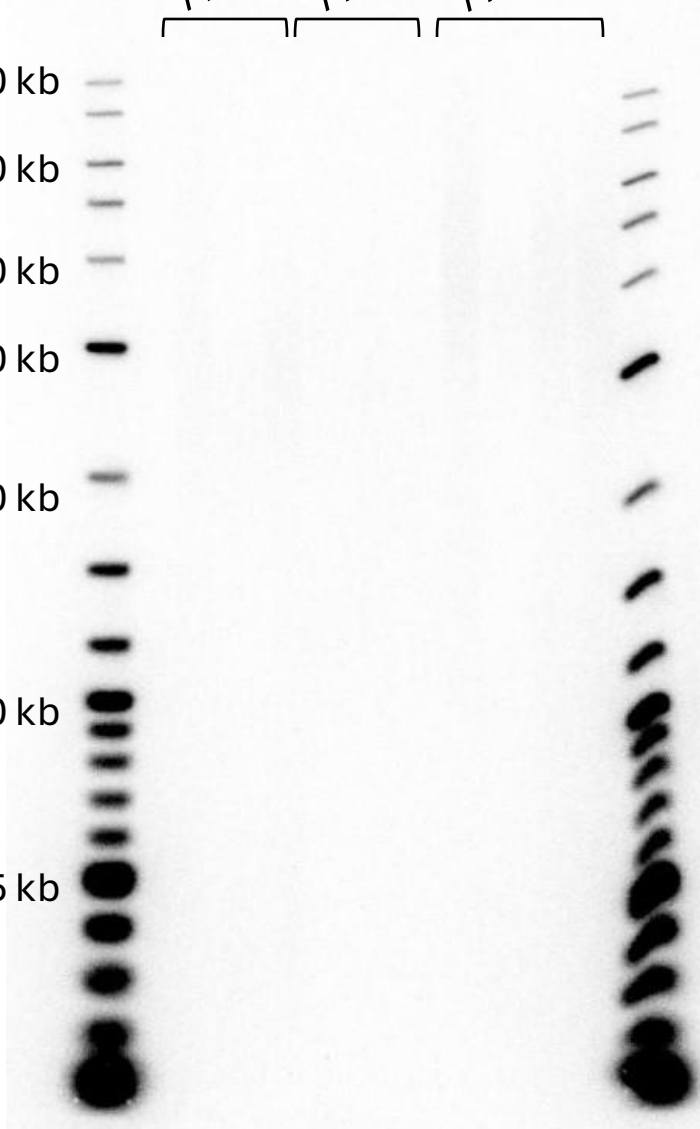

Supplement: S8 Fig — Molecular weight DNA markers (in kb) are shown. (PDF) [file pgen.1011738.s014.pdf]

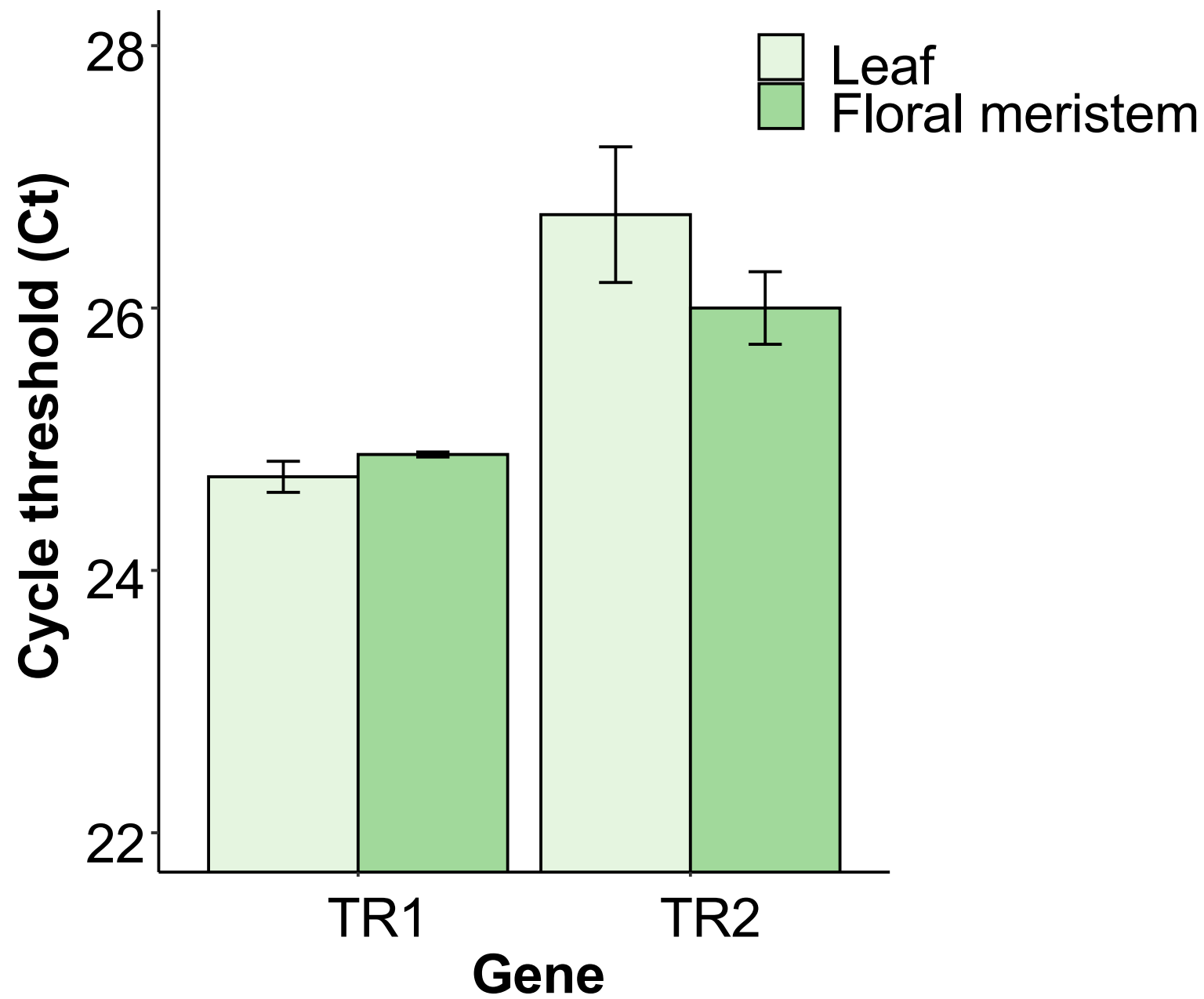

Supplement: S9 Fig — Experiment was conducted with 3 biological replicates. (PDF) [file pgen.1011738.s015.pdf]

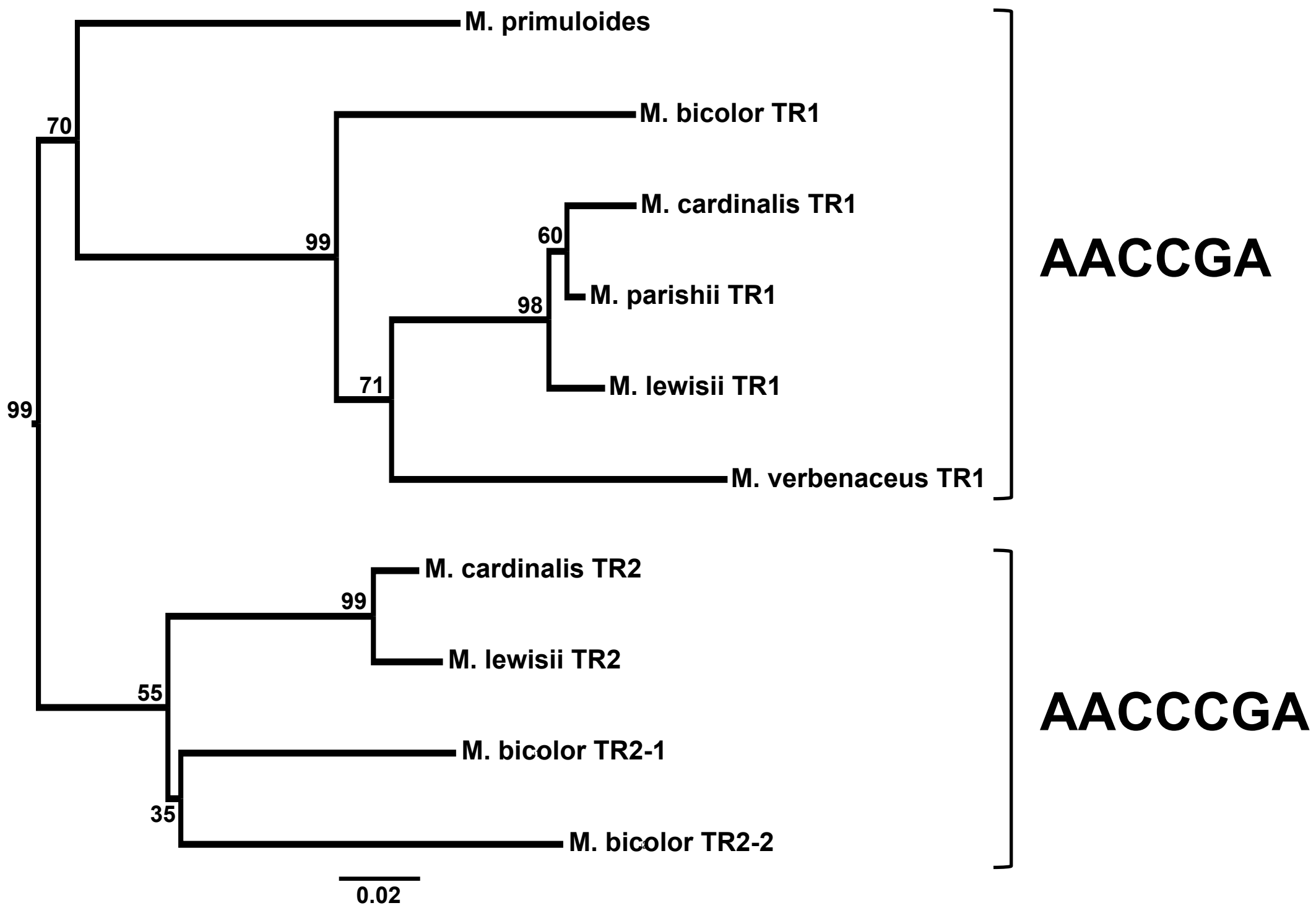

Supplement: S11 Fig — The TR gene phylogeny shows sequences grouping by paralog. The templating sequence within the TR paralog is shown on the right. Internal nodes represent bootstrap support after 1,000 replicates. (PDF) [file pgen.1011738.s017.pdf]

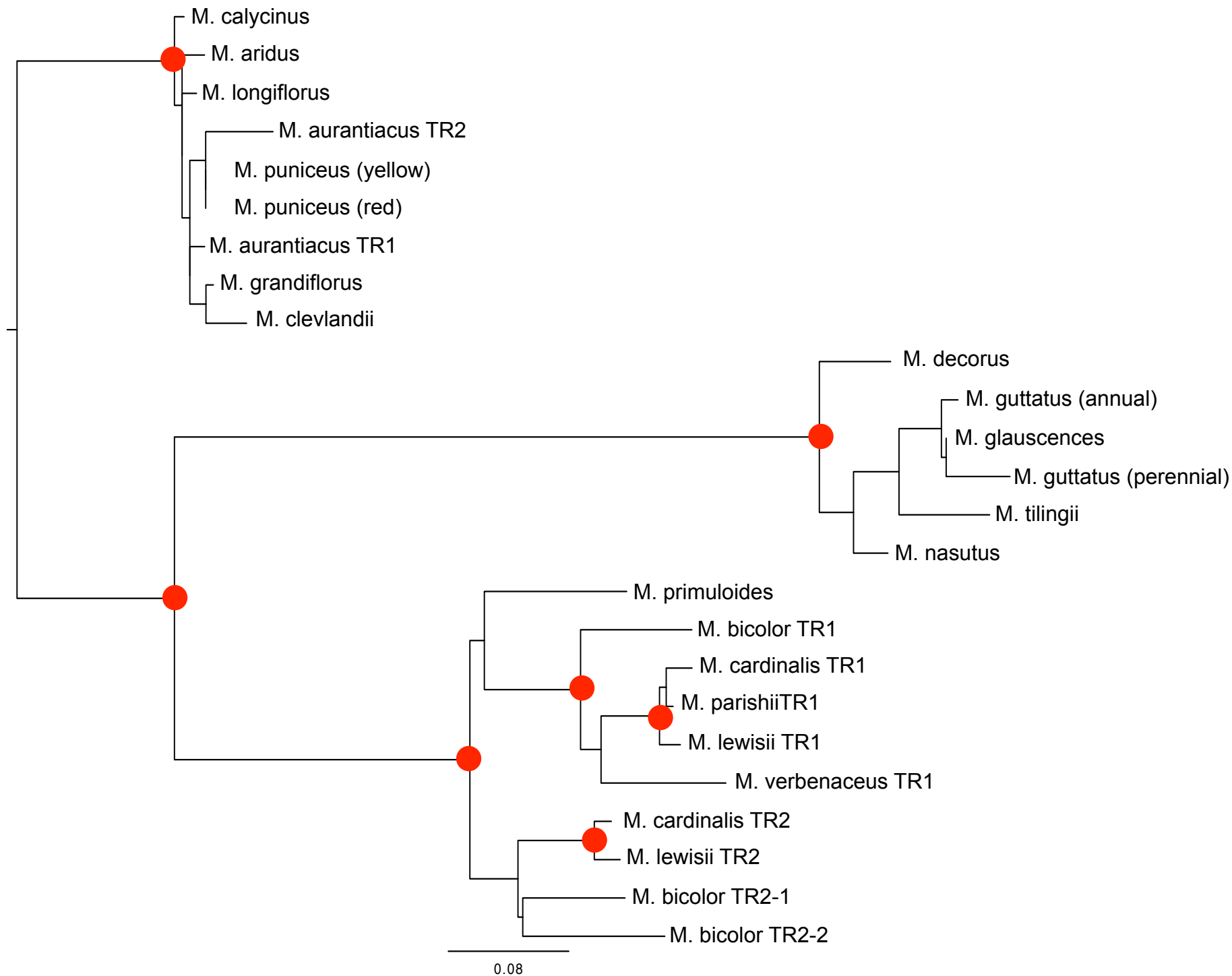

Supplement: S12 Fig — Nodes with bootstrap support >95% are indicated with a red circle. (PDF) [file pgen.1011738.s018.pdf]
