# Supplementary material for: Monkeyflower (Mimulus) uncovers the evolutionary basis of the eukaryote telomere sequence variation
Source: PLoS Genet. 2025 Jun 16;21(6):e1011738. doi: 10.1371/journal.pgen.1011738 (PMC12169523; doi:10.1371/journal.pgen.1011738)
Supplement: S3 Fig — (PDF) [file pgen.1011738.s009.pdf]

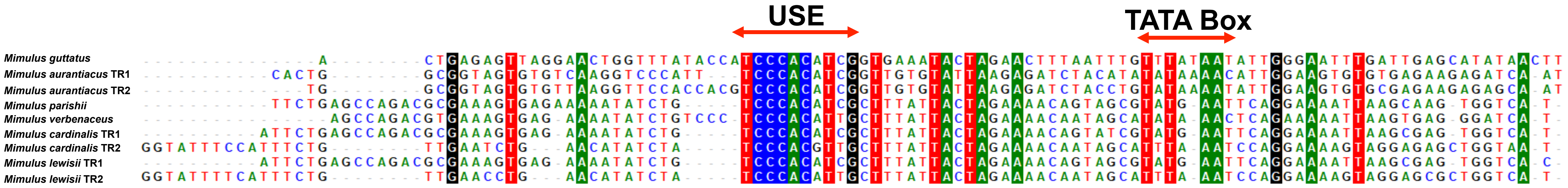
USE
TATA Box
Mimulus guttatus
Mimulus aurantiacus TR1
Mimulus aurantiacus TR2
Mimulus parishii
Mimulus verbenaceus
Mimulus cardinalis TR1
Mimulus cardinalis TR2
Mimulus lewisii TR1
Mimulus lewisii TR2
