# Supplementary material for: Monkeyflower (Mimulus) uncovers the evolutionary basis of the eukaryote telomere sequence variation
Source: PLoS Genet. 2025 Jun 16;21(6):e1011738. doi: 10.1371/journal.pgen.1011738 (PMC12169523; doi:10.1371/journal.pgen.1011738)
Supplement: S4 Fig — Genomic DNA is shown as a positive control. (PDF) [file pgen.1011738.s010.pdf]

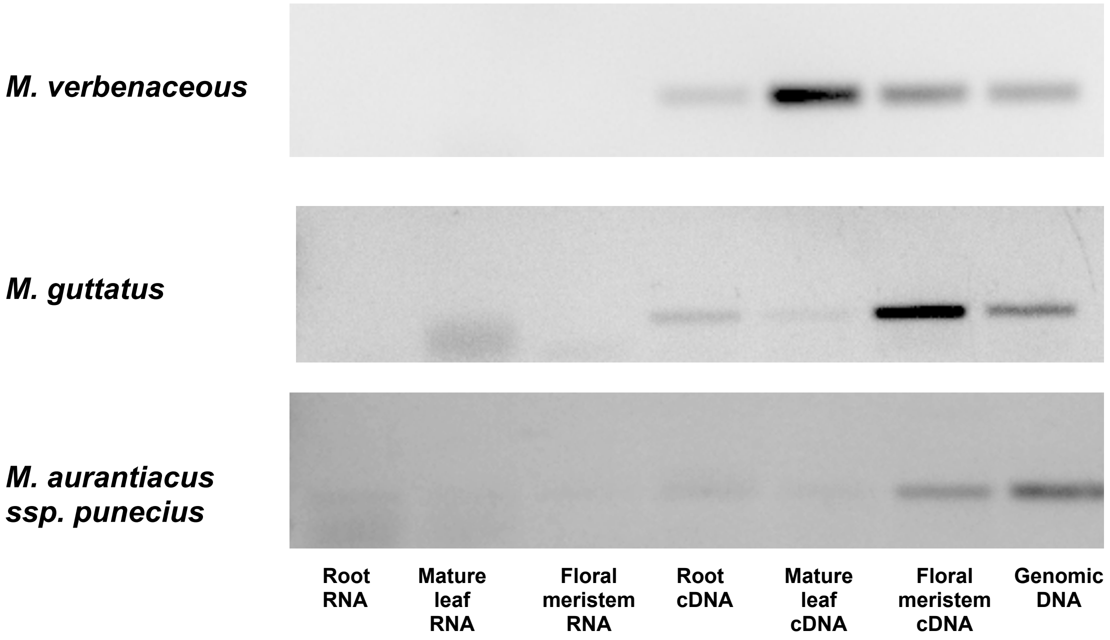
M. verbenaceous
M. guttatus
M. aurantiacus ssp. punecius
Root RNA
Mature leaf RNA
Floral meristem RNA
Root cDNA
Mature leaf cDNA
Floral meristem cDNA
Genomic DNA
