# Supplementary material for: Monkeyflower (Mimulus) uncovers the evolutionary basis of the eukaryote telomere sequence variation
Source: PLoS Genet. 2025 Jun 16;21(6):e1011738. doi: 10.1371/journal.pgen.1011738 (PMC12169523; doi:10.1371/journal.pgen.1011738)
Supplement: S6 Fig — Genomic DNA is shown as a positive control. (PDF) [file pgen.1011738.s012.pdf]

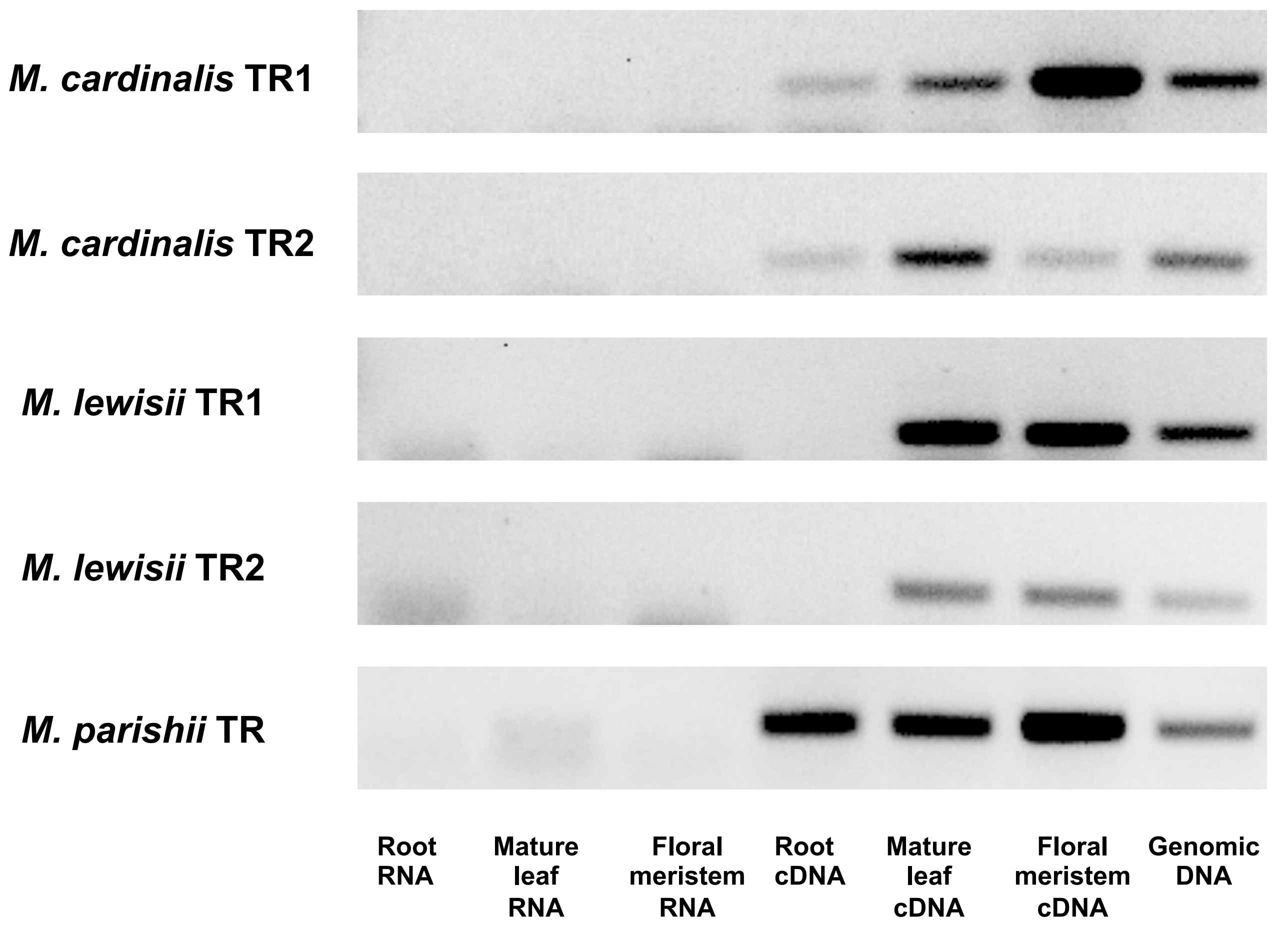
M. cardinalis TR1
M. cardinalis TR2
M. lewisii TR1
M. lewisii TR2
M. parishii TR
Root RNA
Mature leaf RNA
Floral meristem RNA
Root cDNA
Mature leaf cDNA
Floral meristem cDNA
Genomic DNA
