# Supplementary material for: Monkeyflower (Mimulus) uncovers the evolutionary basis of the eukaryote telomere sequence variation
Source: PLoS Genet. 2025 Jun 16;21(6):e1011738. doi: 10.1371/journal.pgen.1011738 (PMC12169523; doi:10.1371/journal.pgen.1011738)
Supplement: S7 Fig — For each species a random nanopore reads was chosen to display the DNA sequence corresponding to the telomere region. GG nucleotides are highlighted in green while GGG nucleotides are highlighted in red. TTTC sequence is highlighted in yellow. (PDF) [file pgen.1011738.s013.pdf]

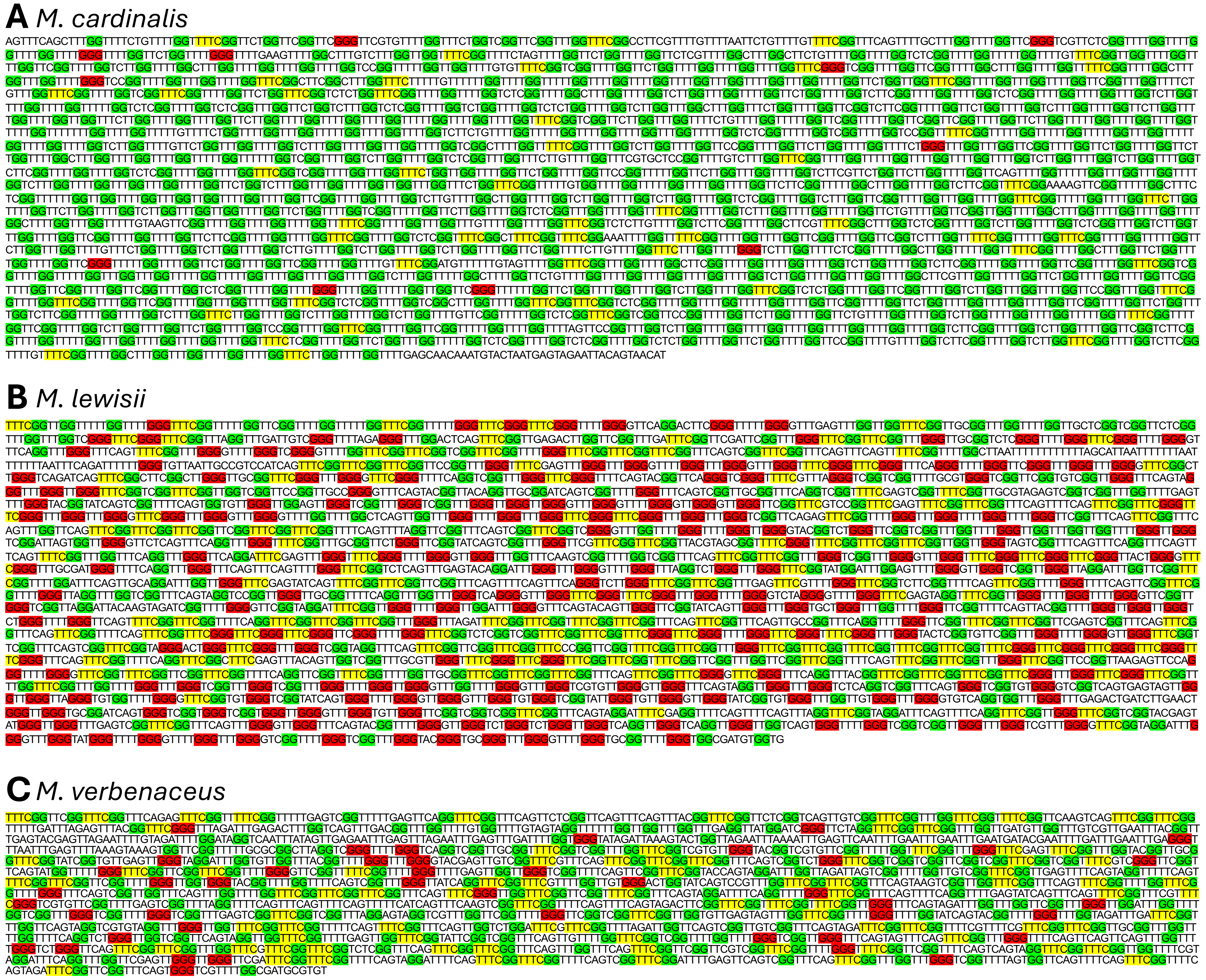
A M. cardinalis
B M. lewisii
C M. verbenaceus
