# Supplementary material for: Monkeyflower (Mimulus) uncovers the evolutionary basis of the eukaryote telomere sequence variation
Source: PLoS Genet. 2025 Jun 16;21(6):e1011738. doi: 10.1371/journal.pgen.1011738 (PMC12169523; doi:10.1371/journal.pgen.1011738)
Supplement: S10 Fig — The TR chromosomal location is indicated with a red arrow. Orange arrows indicate genes and gray boxes indicate orthology between genes. We show the five genes upstream and downstream with orthology. (PDF) [file pgen.1011738.s016.pdf]

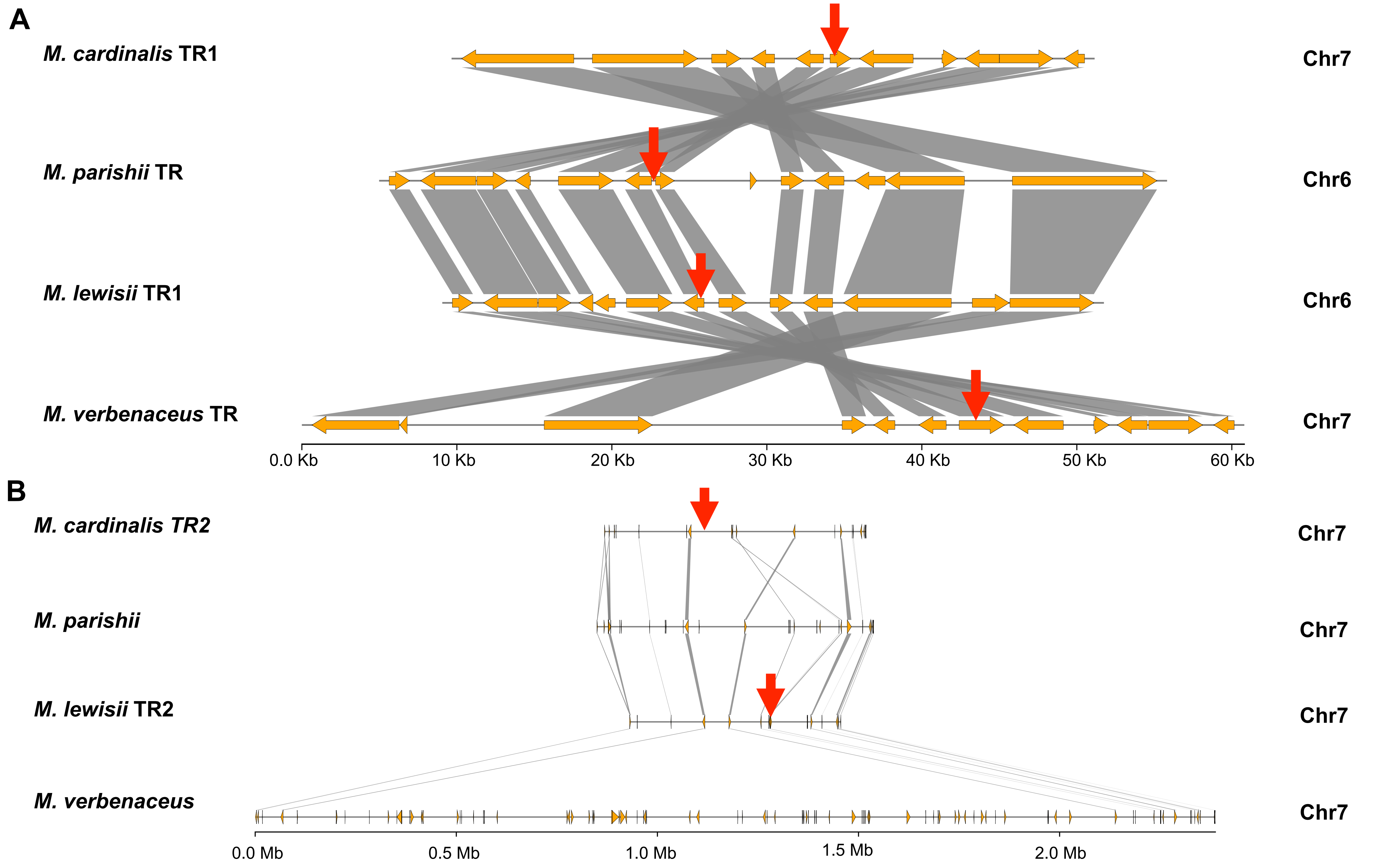
A
M. cardinalis TR1
Chr7
M. parishii TR
Chr6
M. lewisii TR1
Chr6
M. verbenaceus TR
Chr7
0.0 Kb
10 Kb
20 Kb
30 Kb
40 Kb
50 Kb
60 Kb
B
M. cardinalis TR2
Chr7
M. parishii
Chr7
M. lewisii TR2
Chr7
M. verbenaceus
Chr7
0.0 Mb
0.5 Mb
1.0 Mb
1.5 Mb
2.0 Mb
